# Pain and Opioid Consumption After Laparoscopic Versus Open Gastrectomy for Gastric Cancer: A Secondary Analysis of a Multicenter Randomized Clinical Trial (LOGICA-Trial)

**DOI:** 10.1007/s11605-023-05728-3

**Published:** 2023-07-18

**Authors:** Arjen van der Veen, Mark Ramaekers, Marije Marsman, Hylke J. F. Brenkman, Maarten F. J. Seesing, Misha D. P. Luyer, Grard A. P. Nieuwenhuijzen, Jan H. M. B. Stoot, Juul J. W. Tegels, Bas P. L. Wijnhoven, Wobbe O. de Steur, Ewout A. Kouwenhoven, Eelco B. Wassenaar, Werner A. Draaisma, Suzanne S. Gisbertz, Donald L. van der Peet, Anne M. May, Jelle P. Ruurda, Richard van Hillegersberg, LOGICA study group, Leonie Haverkamp, Jeroen E. H. Ponten, Fanny F. B. M. Heesakkers, Karel W. E. Hulsewe, Thais T. T. Tweed, Sjoerd M. Lagarde, Jan J. B. van Lanschot, Henk H. Hartgrink, Marc J. van Det, Peter van Duijvendijk, Edwin S. van der Zaag, Ivo A. M. J. Broeders, Mark I. van Berge Henegouwen, Freek Daams

**Affiliations:** 1 Department of Surgery, University Medical Center Utrecht, Utrecht University, Heidelberglaan 100 G04.228, 3508 GA Utrecht, Netherlands; 2 Department of Surgery, Catharina Hospital, Eindhoven, Netherlands; 3 Department of Anesthesiology, University Medical Center Utrecht, Utrecht University, Utrecht, Netherlands; 4 Department of Surgery, Zuyderland Medical Center, Heerlen and Sittard-Geleen, Netherlands; 5 Department of Surgery, Erasmus University Medical Center, Rotterdam, Netherlands; 6 Department of Surgery, Leiden University Medical Center, Leiden, Netherlands; 7 Department of Surgery, ZGT Hospitals, Almelo, Netherlands; 8 Department of Surgery, Gelre Hospitals, Apeldoorn, Netherlands; 9 Department of Surgery, Meander Medical Center, Amersfoort, Netherlands; 10 Department of Surgery, Amsterdam UMC, Location AMC, University of Amsterdam, Cancer Center Amsterdam, Amsterdam, Netherlands; 11 Department of Surgery, Amsterdam UMC, Location VUmc, University of Amsterdam, Cancer Center Amsterdam, Amsterdam, Netherlands; 12 University Medical Center Utrecht, Utrecht University, Julius Center for Health Sciences and Primary Care, Utrecht, Netherlands

**Keywords:** Gastric cancer, Surgery, Gastrectomy, Laparoscopic gastrectomy, Pain, Opioid consumption, Randomized trial

## Abstract

**Background:**

Laparoscopic gastrectomy could reduce pain and opioid consumption, compared to open gastrectomy. However, it is difficult to judge the clinical relevance of this reduction, since these outcomes are reported in few randomized trials and in limited detail.

**Methods:**

This secondary analysis of a multicenter randomized trial compared laparoscopic versus open gastrectomy for resectable gastric adenocarcinoma (cT1-4aN0-3bM0). Postoperative pain was analyzed by opioid consumption in oral morphine equivalents (OME, mg/day) at postoperative day (POD) 1–5, WHO analgesic steps, and Numeric Rating Scales (NRS, 0–10) at POD 1–10 and discharge. Regression and mixed model analyses were performed, with and without correction for epidural analgesia.

**Results:**

Between 2015 and 2018, 115 patients in the laparoscopic group and 110 in the open group underwent surgery. Some 16 patients (14%) in the laparoscopic group and 73 patients (66%) in the open group received epidural analgesia. At POD 1–3, mean opioid consumption was 131, 118, and 53 mg OME lower in the laparoscopic group, compared to the open group, respectively (all *p* < 0.001). After correcting for epidural analgesia, these differences remained significant at POD 1–2 (47 mg OME, *p* = 0.002 and 69 mg OME, *p* < 0.001, respectively). At discharge, 27% of patients in the laparoscopic group and 43% patients in the open group used oral opioids (*p* = 0.006). Mean highest daily pain scores were between 2 and 4 at all PODs, < 2 at discharge, and did not relevantly differ between treatment arms.

**Conclusion:**

In this multicenter randomized trial, postoperative pain was comparable between laparoscopic and open gastrectomy. After laparoscopic gastrectomy, this was generally achieved without epidural analgesia and with fewer opioids.

**Trial Registration:**

NCT02248519.

**Supplementary Information:**

The online version contains supplementary material available at 10.1007/s11605-023-05728-3.

## Introduction

Gastric cancer is the sixth most prevalent cancer and the third most common cause of cancer-related death worldwide.^1^ Gastrectomy with lymphadenectomy is the cornerstone of multimodality curative treatment.^2^ Open gastrectomy has long been the gold standard worldwide. However, laparoscopic gastrectomy for advanced gastric cancer is rapidly being adopted.^3–5^ Laparoscopic surgery has the potential to reduce pain and thus postoperative opioid consumption.^6,7^ This could be highly relevant since postoperative opioid usage is a potential important contributor to the current opioid epidemic.^8–13^

The Dutch LOGICA-trial on laparoscopic versus open gastrectomy for gastric cancer has reported similar safety and oncological efficacy for laparoscopic and open gastrectomy, in concordance with previous trials from the East.^14–18^ However, detailed pain and analgesic results from randomized trials on laparoscopic versus open gastrectomy are limited. Three Eastern trials on distal gastrectomy indicated a reduction in pain and/or use of analgesics after laparoscopic compared to open gastrectomy.^17,19,20^ However, these trials provided limited details, since analgesic consumption was generally expressed as one composite endpoint (i.e., any analgesics given during POD 6–10 [yes/no]). Hence, it is difficult to judge the clinical relevance of these results for the patient. Furthermore, these trials did not include total gastrectomy.

Postoperative pain was a prespecified outcome measurement during the LOGICA-trial.^21,22^ However, it was not yet reported since data on analgesics and opioid consumption were still lacking, rendering it impossible to present the data in a meaningful manner. The current study aims to provide a detailed secondary analysis, comparing postoperative pain and opioid consumption between laparoscopic and open gastrectomy in the multicenter randomized LOGICA-trial.^14^ It was hypothesized that laparoscopic gastrectomy would lead to reduced pain and/or reduced opioid consumption.

## Methods

### LOGICA-Trial Design and Previous Results

All patients who participated in the LOGICA-trial were included in this secondary analysis. The LOGICA-trial was a multicenter randomized controlled, open-label, superiority trial comparing laparoscopic with open gastrectomy in 10 Dutch hospitals. The study protocol was approved by the institutional review board at each participating hospital, and all patients signed written informed consent. The protocol and main results were published previously (clinicaltrials.gov NCT02248519).^14,21^ Briefly, between 2015 and 2018, 227 patients with surgically resectable (cT1-4aN0-3bM0) gastric cancer were included and randomized to laparoscopic (*n* = 115) or open gastrectomy (*n* = 112). Both groups did not differ regarding median initial hospital stay (7 versus 7 days, *p* = 0.34), postoperative complication rate (44% versus 42%, *p* = 0.91), and all other postoperative outcome parameters.

### Postoperative Protocol

As previously described, multiple quality control measures were included in the trial, and the treatment protocols were in accordance with the guidelines for Enhanced Recovery After Surgery (ERAS).^14,21,23^

Postoperative pain protocols were left to the discretion of each participating hospital and did not differ between treatment arms, except for epidural analgesia. For open gastrectomy, epidural analgesia was the standard unless there were (relative) contraindications. For laparoscopic gastrectomy, epidural analgesia was not allowed according to the trial protocol, and pain control was achieved via intravenous opioids, oral opioids, or paracetamol only. Patients that received epidural analgesia anyway were regarded protocol-violations but analyzed according to the intention-to-treat principle nonetheless. Epidurals were placed between intervertebral levels T5–T10. All infusions contained local anesthetics (all hospitals used bupivacaine) and an opioid, since the combination of local anesthetics with opioids provides superior analgesia and is thus recommended in recognized guidelines.^24^ The type of opioid and infusion rates varied between hospitals. All hospitals administrated paracetamol 1000 mg/6 h, but nonsteroidal anti-inflammatory drug (NSAID) usage was limited. Between hospitals, different opioids were used orally and intravenously, and intravenous opioids were administered in different ways (as single injections, continuous administration, and/or patient-controlled boluses). Some hospitals added esketamine as part of a multimodal analgesic protocol in patients with insufficient pain control from opioids.

### Postoperative Evaluation and Pain Control

Standardly, pain scores (NRS) were assessed by the ward nurse once every 8 h and after each intervention for pain. Additionally, a dedicated pain team evaluated pain control at POD 1 in all patients and hereafter daily in patients receiving epidural analgesia, intravenous opioids, or patients in whom pain control was difficult. This pain team evaluated pain scores in combination with opioid consumption, side effects, and complications, and in case of epidural analgesia, the epidural sensory block range was tested. An NRS < 4 in rest and < 6 while mobilizing was generally considered to be acceptable. In case of insufficient pain control with opioids, analgesics daily opioid dose was increased, or non-opioids were added (for example, NSAIDs or esketamine). In case of epidural analgesia with an inadequate sensory block, an epidural top-up was performed, and continuous infusion was increased if a top-up was successful. If a top-up was unsuccessful, the epidural was removed and the patient switched to intravenous or oral opioids. Opioids were removed from the epidural mixture in patients who received opioids parallel to epidural analgesia. In case of sufficient pain control, intravenous opioids or epidural analgesia was gradually switched to oral opioids and then to paracetamol only.

### Primary Outcomes

The primary outcomes of the current study included daily postoperative pain scores, daily analgesic steps of the WHO pain ladder as an indicator of pain severity, and daily opioid consumption.^25–27^

Pain scores were assessed in admitted patients at POD 1–10 and at the morning of discharge. Pain was assessed on a 0–10 NRS.^22^ The mean of the highest collected NRS pain scores of the day were used for the main analyses.

Analgesic steps were assessed in admitted patients at POD 1–10 and at the day of discharge. Analgesic steps were based on the WHO analgesic ladder: (I) no analgesics or paracetamol ± NSAID, (II) addition of weak opioids (i.e., tramadol), (III) addition of strong opioids, and (IV) addition of epidural or esketamine.^25–27^ For illustrative purposes, step III was split by route of administration: orally or intravenously.

Data on all administered analgetics, administration routes, and dosages were collected for postoperative day (POD) 1–5. For optimal comparison, opioids were converted into daily oral morphine equivalents (OME),^28,29^ for example, 1 mg intravenous (IV) morphine = 3 mg OME.

### Secondary Outcomes

Secondary outcomes included quality and efficacy of epidural analgesia: quality of sensory block, incidence of top-ups, replacements, need for additional analgesia, day of removal, and occurrence of minor or major epidural-related complications (Supplementary material 1).

Further secondary outcomes included addition of nonsteroidal anti-inflammatory drugs (NSAIDs) or esketamine, opioid intoxications, use of an enema, and mobilization milestones (first time sitting in a chair or walking in the hallway).

### Data Collection

Analgesic steps and pain severity scores at POD 1–5 were registered prospectively in the LOGICA electronic case report forms (eCRF). An additional retrospective data collection was performed in each participating hospital’s patient files and medication dispense registries, to collect the data regarding opioid consumption (including dosages) at POD 1–5, analgesic steps, and pain severity scores at POD 6–10 and at discharge and all secondary outcomes. Opioid consumption was not collected after POD 5, since this retrospective data collection was time-consuming.

### Statistical Considerations

This was a secondary analysis of the LOGICA-trial. NRS pain scores were a prespecified outcome measurement, whereas opioid consumption was not.^21^ Analyses were according to intention-to-treat.^14,21^ Primary outcomes were displayed descriptively in bar and line charts. Additionally, comparative statistics were performed between treatment arms. Differences in pain scores and daily opioid consumption at POD 1–5 were analyzed with linear mixed-effects models. In these longitudinal analyses, between group differences were reported for each of the dependent time points at POD 1–5. Pain at discharge was analyzed with linear regression, and analgesic step at discharge was analyzed with Poisson regression with robust error variances for binary outcomes.^30,31^ Length of stay until discharge did not vary between the laparoscopic and open group, but did vary within both groups; hence, it was mathematically not feasible to analyze these endpoints in a mixed model. The study protocol caused an inherent difference between treatment arms in epidural analgesia and consequently analgesic steps at the first PODs.^21^ Hence, comparative statistics were performed only for the analgesic step at discharge and not at POD 1–10. For optimal transparency and to evaluate possible bias by epidural, all models were performed with and without correction for initiation of epidural analgesia. Secondary outcomes were compared with chi-squared tests, Fisher’s exact tests, or Mann–Whitney U tests,^32^ depending on the type of data and distribution. *p* < 0.05 was considered statistically significant. Supplementary material 2 provides additional methodological details.

## Results

### Primary Outcomes

Between 2015 and 2018, 115 patients in the laparoscopic group and 110 in the open group underwent surgery (Table 1). Supplementary material 3 displays the study flowchart. Epidural analgesia was initiated in 16 patients (14%) in the laparoscopic group and 73 patients (66%) in the open group (Supplementary material 4).

**Table 1:** Type of surgery, analgesics, and secondary outcomes. *NA* not applicable, *IV* intravenous, *IM* intramuscular, *SC* subcutaneous, *NSAID* nonsteroidal anti-inflammatory drug, *POD* postoperative day, *IQR* interquartile range

	Laparoscopic gastrectomy				Open gastrectomy					
*n* (%)	*n* = 115		*Missing or NA*		*n* = 110		*Missing or NA*		*p*	
Type of operation			*0*	*(0)*			*0*	*(0)*	0.397	
Total gastrectomy	48	(41.7)			43	(39.1)				
Distal gastrectomy	59	(51.3)			64	(58.2)				
Esophagogastric resection	1	(0.9)			0	(0.0)				
No resection	7	(6.1)			3	(2.7)				
IV opioid POD 1–5**	62	(57.4)	*7*	*(6.1)*	49	(45.4)	*2*	*(1.8)*		^3^
IV opioid type			*54*	*(47)*			*62*	*(56.4)*		^3^
Piritramide	10	(16.4)			4	(8.3)				
Fentanyl	3	(4.9)			10	(20.8)				
Morphine	48	(78.7)			34	(70.8)				
IM/SC opioid POD 1–5**	24	(23.1)	*11*	*(9.6)*	17	(16.5)	*7*	*(6.4)*		^3^
IM/SC opioid type			*92*	*(80)*			*94*	*(85.5)*		^3^
Piritramide	5	(21.7)			10	(62.5)				
Fentanyl	2	(8.7)			0	(0.0)				
Morphine	16	(69.6)			6	(37.5)				
Oral opioid POD 1–5**	77	(74.0)	*11*	*(9.6)*	89	(81.7)	*1*	*(0.9)*		^3^
Oral opioid type			*46*	*(40)*			*29*	*(26.4)*		^3^
Oxycodone	67	(97.1)			79	(97.5)				
Tramadol	1	(1.4)			2	(2.5)				
Buprenorphine	1	(1.4)			0	(0.0)				
Esketamine POD 1–5**	14	(12.7)	*5*	*(4.3)*	15	(13.8)	*1*	*(0.9)*	0.979	
NSAID POD 1–5**			*4*	*(3.5)*			*1*	*(0.9)*	0.611	^1^
Metamizole	4	(3.6)			6	(5.5)				
Diclofenac	2	(1.8)			1	(0.9)				
Naproxen	0	(0.0)			1	(0.9)				
No	105	(94.6)			101	(92.7)				
Enema POD 1–5**	43	(39.1)	*5*	*(4.3)*	41	(38.0)	*2*	*(1.8)*	0.975	
Opioid intoxication	0	(0.0)	*5*	*(4.3)*	0	(0.0)	*2*	*(1.8)*	NA	
POD of first sitting in chair (median [IQR])	1	[1.00, 1.00]	6	*(5.2)*	1	[1.00, 2.00]	2	*(1.8)*	0.048	^2^
POD of first walking in hallway (median [IQR])	2	[1.00, 3.00]	12	*(10.4)*	2	[2.00, 3.00]	7	*(6.4)*	0.004	^2^

^*^POD 0 = day of surgery

^**^This variable indicates whether the medication was given at least once during the first 5 postoperative days. If such a medication was given, then the type of medication was constant over the different PODs (except for 1 patient who received IV Morphine on POD 1–2 and IV Piritramide on POD 4, registered here under IV Morphine)

^1^Fisher’s exact test performed

^2^Mann-Whitney U test performed

^3^No statistical test performed

**Fig. 1:**
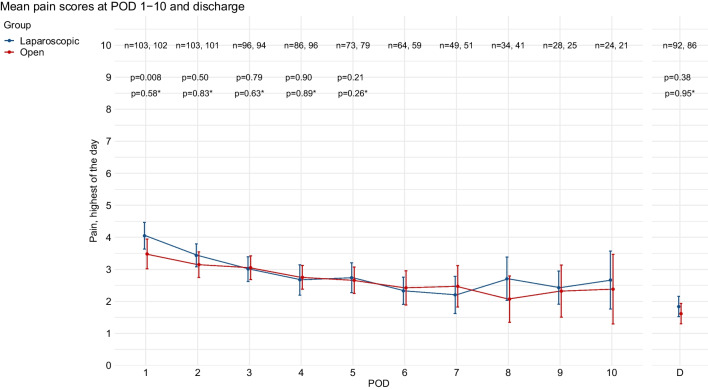
Mean pain scores (highest of the day) at POD 1–10 and discharge, with 95% confidence intervals. *p*-values from the mixed model between group comparison at POD 1–5 and linear regression at discharge (Table 2) are displayed above the brackets. * = *p*-value corrected for epidural analgesia. POD, postoperative day; D, day of discharge; n, number of patients. Of note, day of discharge is variable per patient and often not directly following POD 10

Mean highest daily pain scores during admission at POD 1–10 and at discharge are displayed descriptively in Fig. 1. At POD 1, the highest daily pain score was mean 0.8 point higher in the laparoscopic group, compared to the open group (95% CI [0.20–1.38], *p* = 0.008). After correcting for epidural analgesia, the highest daily pain score at POD 1 did no longer differ between the laparoscopic versus the open group (mean difference 0.20 points, 95% CI [− 0.50 to 0.90], *p* = 0.576). At POD 2–10 and at discharge, there were no significant differences between treatment arms, regardless of correction for epidural analgesia (Table 2). Mean first daily pain scores and median pain scores were generally lower in both treatment arms but showed similar results between treatment arms as the mean highest daily pain scores (Supplementary material 5).

**Table 2:** Pain scores and opioid consumption: between group differences of mixed model and linear regression analyses. The highest pain score of the day was used. The between-group differences are displayed for laparoscopic gastrectomy, compared to open gastrectomy. Analyses are displayed with and without correction of pre-operative initiation of epidural analgesia. In addition, all analyses were corrected for the stratification factors (total/distal gastrectomy and hospital). Bold values indicate significant differences. The number of patients included in the mixed model for total opioids were 110 for the laparoscopic and 100 for the open group. For pain score, this was 106 in the laparoscopic and 109 in the open group. *L* laparoscopic group, *O* open group, *CI* confidence interval, *OME* oral morphine equivalent

	Mixed model – between group differences															Linear regression		
	POD 1			POD 2			POD 3			POD 4			POD 5			Discharge		
	ΔMean [95% CI]		*p*	ΔMean [95% CI]		*p*	ΔMean [95% CI]		*p*	ΔMean [95% CI]		*p*	ΔMean [95% CI]		*p*	ΔMean [95% CI]		*p*
Uncorrected for epidural																		
Pain score	**0.79**	**[0.20 to 1.38]**	**0.008**	0.20	[**− **0.39 to 0.79]	0.501	** − **0.08	[**− **0.70 to 0.54]	0.790	** − **0.04	[**− **0.71 to 0.62]	0.898	− 0.64	[− 1.64 to 0.36]	0.207	0.20	[**− **0.25 to 0.65]	0.379
Total opioid, mg OME	** − 131**	**[− 158 to − 105]**	** < 0.001**	** − 118**	**[− 144 to − 92]**	** < 0.001**	** − 53**	**[− 80 to − 27]**	** < 0.001**	** − **13	[**− **45 to 19]	0.422	− 18	[− 47 to 11]	0.223			
Corrected for epidural																		
Pain score	0.20	[**− **0.50 to 0.90]	0.576	** − **0.08	[**− **0.79 to 0.63]	0.828	** − **0.18	[**− **0.92 to 0.56]	0.630	0.05	[**− **0.72 to 0.83]	0.889	** − **0.63	[**− **1.72 to 0.47]	0.263	0.02	[**− **0.59 to 0.62]	0.949
Total opioid, mg OME	** − 47**	**[− 77 to − 18]**	**0.002**	** − 69**	**[− 98 to − 40]**	** < 0.001**	** − **23	[**− **52 to 6]	0.120	5	[**− **30 to 39]	0.782	** − **8	[**− **40 to 24]	0.625			

**Fig. 2:**
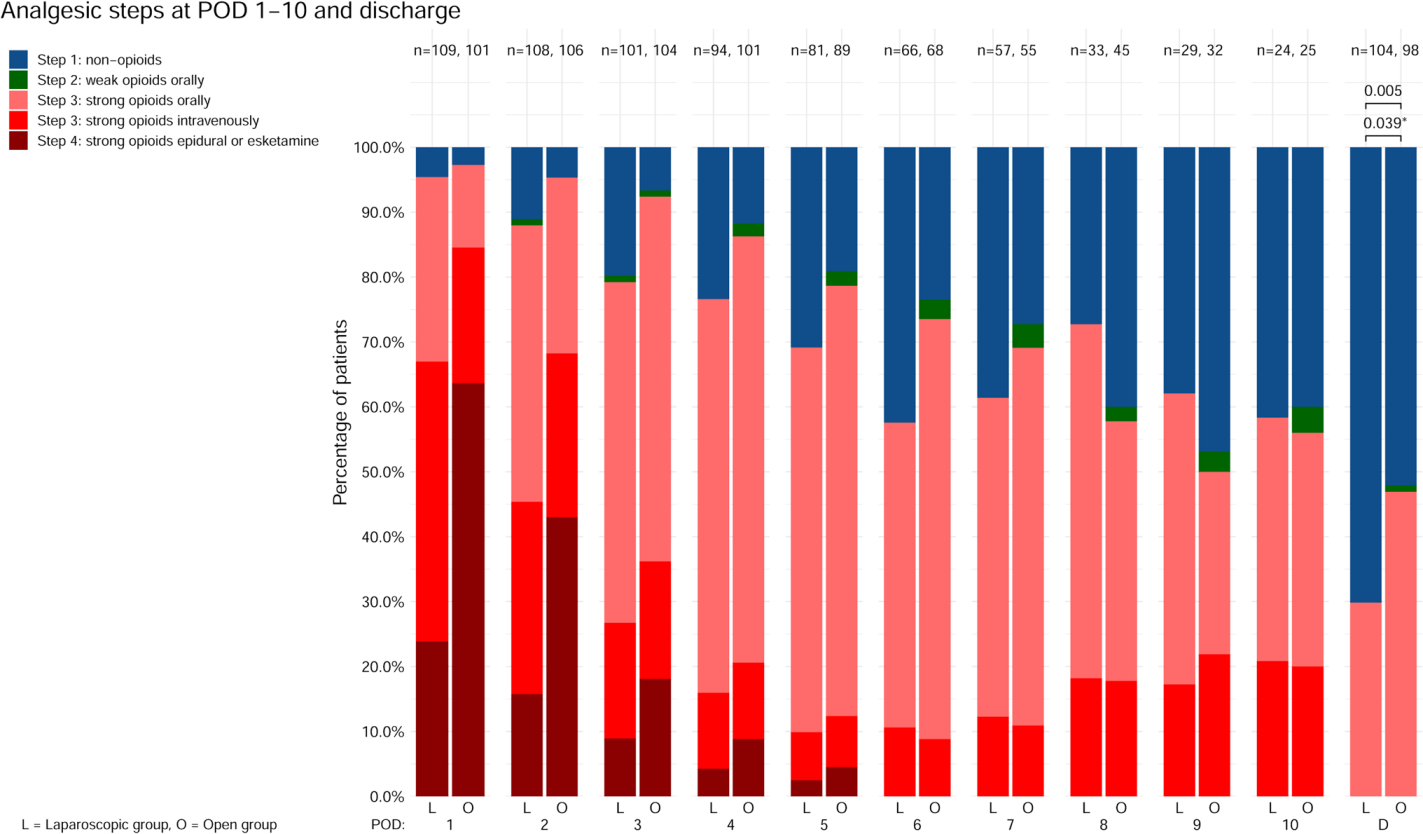
Analgesic steps at POD 1–10 during hospital admission and at discharge. Of note, day of discharge is variable per patient and often not directly following POD 10. *p*-values from the Poisson regressions are displayed above the brackets. * = *p*-value corrected for epidural analgesia. L, laparoscopic group; O, open group; POD, postoperative day; D, day of discharge; n, number of patients

The analgesia use, as WHO pain ladder steps during admission at POD 1–10 and at discharge, is displayed descriptively in Fig. 2. At POD 1–7, step 1 analgesics were more often administrated in the laparoscopic group, compared to the open group, who received more often step 3 analgesics. At POD 8–10, the majority of laparoscopic patients had been discharged, and this difference was no longer present. Step 2 analgetics (weak opioids) were seldom prescribed. At discharge, step 2–3 analgesics were administered in 27% of patients in the laparoscopic group versus 43% of patients in the open group (RR 0.88, 95% CI [0.80–0.96], *p* = 0.005) (Fig. 2). This difference remained significant after correcting for previous epidural analgesia (RR 0.89, 95% CI [0.80–0.99], *p* = 0.039).

**Fig. 3:**
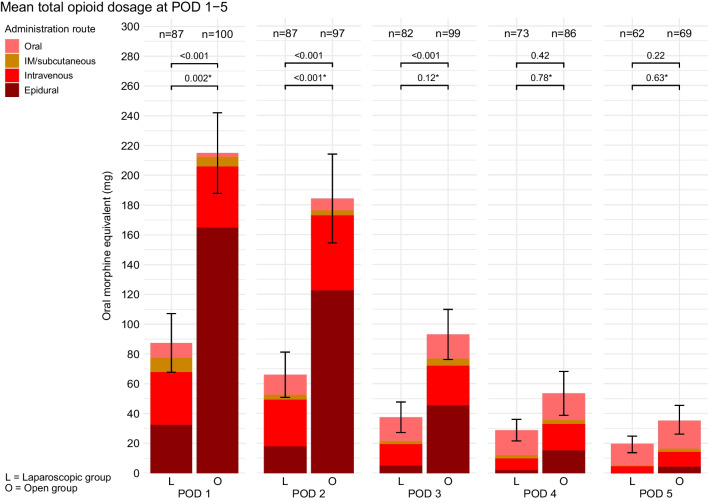
Mean total opioid dosages at POD 1–5. 95% confidence intervals are displayed for the mean total opioid dosages (the sum of the 4 administration routes). *p*-values from the mixed model between group comparison (Table 2) are displayed above the brackets. * = *p*-value corrected for epidural analgesia. L, laparoscopic group; O, open group; POD, postoperative day; IM, intramuscular; n, number of patients

Mean daily opioid consumption per administration route is displayed descriptively in Fig. 3. At POD 1–3, mean daily total opioid consumptions were 131, 118, and 53 mg OME lower in the laparoscopic group, compared to the open group, respectively (95% CI [− 158 to − 105], *p* < 0.001; 95% CI [− 144 to − 92], *p* < 0.001; and 95% CI [− 80 to − 27], *p* < 0.001, respectively) (Table 2). At POD 4–5, there were no significant differences between treatment arms (Table 2). After correcting for epidural analgesia, mixed model-estimated mean total opioid consumption at POD 1 and 2 were 47 and 69 mg OME lower in the laparoscopic group, compared to the open group, respectively (95% CIs [− 77 to − 18] and [− 98 to − 40], *p* = 0.01 and *p* < 0.001, respectively), whereas POD 3–5 did not significantly differ between treatment arms (Table 2).

### Secondary Outcomes

Secondary outcomes regarding quality and efficacy of epidural analgesia are displayed in Table 1 and Supplementary material 4. Epidural analgesia resulted in an adequate sensible block in 78–100% of patients. However, this could be an overestimation, as patients with an inadequate block and subsequently removed epidural could have been reported as missing/not applicable (Supplementary material 4). Most epidurals were removed at POD 2 and 3. Of the patients that received epidural analgesia, intravenous opioids were given at least once during POD 1–5 in 21% of the laparoscopic group and 28% of the open group. In 6 out of 73 patients (8%) with an epidural in the open group, hypotension occurred as a (minor) complication. No other epidural-related complications were reported.

The use of postoperative esketamine, NSAIDs, and postoperative enema did not differ between treatment arms (Table 1). No opioid intoxications occurred.

The probabilities of earlier first time sitting in a chair and walking in the hallway were higher in the laparoscopic group, compared to the open group (estimated probabilities 0.56, 95% CI 0.50–0.61, *p* = 0.048 and 0.61, 95% CI 0.54–0.68, *p* = 0.0041, respectively). However, median POD and interquartile ranges (IQR) were low in both arms for first time sitting in a chair (median 1 [IQR 1–1] versus 1 [IQR 1–2]) and walking in the hallway (median 2 [IQR 1–3] versus 2 [IQR 2–3]).

### Per-protocol Analyses

All analyses were repeated in the prespecified per-protocol dataset (n=106 versus n=105, Supplementary material 3), and no relevant differences were found compared to the main intention-to-treat dataset.

## Discussion

In this multicenter randomized trial on laparoscopic versus open gastrectomy for gastric cancer, pain scores were comparable and acceptable in both treatment arms during all PODs and at discharge (between 2 and 4 at all PODs and < 2 at discharge). Mobilization milestones were quickly reached in both treatment arms and only modestly quicker in the laparoscopic group. In the laparoscopic group, mean daily opioid consumption was significantly lower, and significantly fewer patients used oral opioids at discharge. Hence, laparoscopic gastrectomy led to adequate pain control, generally without epidural analgesia and with a clinically relevantly lower consumption of opioids, compared to open gastrectomy.

The higher opioid consumptions in the open group were partly due to the majority of this group receiving epidural analgesia, through which local anesthetics and opioids are administered. Epidural administered opioids also reach the systemic circulation and were therefore converted into daily oral morphine equivalents (via recognized conversion values) and added to the daily opioid consumption.^28,29,33^ Nevertheless, even after correcting for epidural analgesia, mean daily opioid consumption at POD 1–2 was still up to 69 mg OME lower in the laparoscopic group, which likely reflects lower analgesic requirements due to reduced pain from the smaller incisions of the laparoscopic surgery itself. Furthermore, usage of oral opioids at POD 1–7 and discharge (27% versus 43%) was lower in the laparoscopic group, compared to the open group.

These opioid reductions are deemed especially relevant in light of the current opioid epidemic.^12,13,34^ In the USA, approximately 76 million adults reported to have used prescribed opioid drugs in 2015–2016, and prescription opioid deaths have increased from 3442 deaths in 1999 to 17,029 in 2017.^34^ In Europe and more specifically the Netherlands, prescription opioid users nearly doubled from 4109 per 100,000 inhabitants in 2008 to 7489 in 2017.^8^ Oxycodone use almost quadrupled in this period, and opioid prescribing after surgery, especially in the context of increasingly short hospital stays due to ERAS protocols, has been recognized as an important potential contributor to opioid misuse and related harm.^8,10^ Hence, the lower opioid consumption at discharge in the laparoscopic surgery group could be a relevant benefit.

It would be especially relevant if this would also result in reduced long-term opioid users after laparoscopic gastrectomy. Chronic opioid use often begins with treatment of acute pain, and approximately 3.3% of patients exposed to chronic use become addicted.^8,9^ Indeed, 3 recent non-randomized studies evaluated laparoscopic versus open colectomy, and 2 of these studies associated laparoscopic surgery with both reduced short-term and long-term opioid usage.^6,7,35^ Unfortunately, the current trial only had data up to 1-year postoperatively regarding patient-reported pain scores (showing no differences between treatment arms), but no data on opioid consumption up to 1-year postoperatively.^14^ Future research is required to examine how many short-term opioid users become long-term users after gastrectomy and whether this differs between laparoscopic and open gastrectomy.

Three trials on distal gastrectomy briefly reported on pain or analgesic consumption upon publishing the main trial results.^17,19,20^ However, none of these trials reported detailed descriptions of the postoperative pain protocols. Importantly, opioid dosages in morphine equivalents per postoperative day were not reported. Instead, one or two composite outcomes were included with limited details (i.e., any analgesics given during POD 6–10 [yes/no]). Although this makes it hard to judge the clinical relevance of these outcomes for the patient, these composite outcomes did indicate reduced pain and/or analgesics after laparoscopic gastrectomy, which is in line with the current study results. An advantage of the current study is that the pain and analgesic-related data were reported in a high level of detail and that total gastrectomy was also included.

Epidural analgesia is an invasive procedure, and complications can occur, such as hypotension and not adequately functioning epidural catheters in up to one-third of patients.^23,36^ Fortunately, complications such as hypotension were only reported in a minority of patients in the current trial, though this might be an underrepresentation due to the retrospective data collection of epidural details.^37^ Importantly, 29% of patients in the open group with epidural analgesia also received intravenous opioids sometime during the first 5 PODs, indicating that the epidural analgesia itself often was insufficient. Nevertheless, adequate pain control was achieved in both treatment arms.

An important limitation of the current study is that the trial protocol only allowed for epidural analgesia in the open group, since ERAS guidelines indicated that epidural analgesia provided superior pain control compared to intravenous analgesia in open abdominal surgery.^23^ In the laparoscopic group, it was hypothesized that adequate pain control could be achieved without epidural analgesia. To address possible bias by epidural, analyses were performed with and without correction for epidural analgesia. Furthermore, we reported oral opioid consumption at discharge (> 90% of epidurals were removed at POD 1–3, median day of hospital discharge was POD 7, Fig. 2). Though selection bias could remain, these end points indicate reduced analgesia in the laparoscopic group. Furthermore, the acceptable postoperative pain scores in the laparoscopic group confirm our hypothesis that this operation can be performed without epidural analgesia. A further limitation is that protocol violations occurred in 11% of the laparoscopic group that received epidural analgesia regardless. These were caused at random due to logistical errors, mainly the responsible anesthesiologist not being aware of the trial protocol. Presumably this did not affect our conclusions, since analyses were performed according to intention-to-treat and were performed with and without correction for epidural analgesia. An additional limitation is that clinicians were not blinded for the randomization. Although pain scores at discharge were comparable between treatment arms, clinician bias could theoretically have contributed to a difference in opioids prescribed at discharge. Lastly, a limitation is that the postoperative pain management protocols differed between hospitals. However, aside from epidural analgesia, in each hospital, the protocols did not differ between treatment arms, and randomization was stratified by hospital. Hence, this presumably did not affect our conclusions and, as it reflects daily practice, allows for increased generalizability of the current trial results to the general population.

Strengths of the current study are that it is the first randomized trial on this subject in a Western population and the first to also include total gastrectomy.^18^ Length of hospital stay did not differ between laparoscopic and open gastrectomy, which allowed for a smooth comparison of the study outcomes per postoperative day and at discharge. A pain team was involved in each hospital, and the primary outcomes were presented in a high level of detail. An ERAS protocol and multiple surgical quality control measures were in place, as described previously.^14^

In conclusion, in the current multicenter randomized trial on laparoscopic versus open gastrectomy, adequate pain management was achieved in both treatment arms. After laparoscopic gastrectomy, this was generally achieved without epidural analgesia and with significantly lower consumption of opioids, compared to open gastrectomy.

### Supplementary Information

Below is the link to the electronic supplementary material.

Supplementary file1 (DOCX 550 KB)

Supplementary file2 (PDF 1511 KB)

## Data Availability

Individual patient data is not available.
