# Supplementary material for: Pain and Opioid Consumption After Laparoscopic Versus Open Gastrectomy for Gastric Cancer: A Secondary Analysis of a Multicenter Randomized Clinical Trial (LOGICA-Trial)
Source: J Gastrointest Surg. 2023 Jul 18;27(10):2057–67. doi: 10.1007/s11605-023-05728-3 (PMC10579125; doi:10.1007/s11605-023-05728-3)
Supplement: Supplementary file 1 — Supplementary file1 (DOCX 550 KB) [file 11605_2023_5728_MOESM1_ESM.docx]

**Supplementary material 1 - epidural complication definitions**

The patient charts were retrospectively reviewed for the epidural related complications. Minor complications were defined as: catheter problems, hypotension, bradypnea, transient tingling in the legs, hallucinations and other. Major complications were defined as: meningitis, epidural hematoma, epidural abscess and other.

**Supplementary material 2 – additional methodological details**

The study protocol was approved by the institutional review board at each participating hospital (Haverkamp et al. BMC Cancer. 2015;15:556). This trial was funded by ZonMW (The Netherlands Organisation for Health Research and Development), project number 837002502.

Differences in analgesic step at discharge between treatment arms were analyzed with Poisson regression with robust error variances for binary outcomes, providing relative risks (RR) for having step II-III, instead of step I (Zou et al. Am J Epidemiol. 2004;159(7):702–6, Knol et al. Cmaj. 2012;184(8):895–9). All statistical models were corrected for the stratification factors (total/distal gastrectomy and hospital). For optimal transparency, all models were performed with and without correction for initiation of epidural analgesia for linear and Poisson regression and an interaction factor epidural analgesia*postoperative day for mixed-effects models. Modelling assumptions were examined and met. The amount of missing and non-applicable (NA) data was reported. In the linear mixed-effects models, only pain scores at POD 1-5 were included, since the score at POD 6-10 were often missing non-randomly due to discharged patients. Furthermore, missing data of patients who died or were discharged within 5 days postoperatively were regarded as non-random and this was corrected for by adding this missing pattern as an interaction variable (Son et al. “Application of pattern mixture models to address missing data in longitudinal data analysis using spss”. Nurs Res. 2012;61(3):195–203.). Other missing data were regarded to be at random and were accounted for in the mixed-effects models (with a first-order autoregressive structure with homogenous variances) and excluded from the other analyses. Mixed model analyses were performed using IBM SPSS Statistics version 26.0.0.1 (IBM Corp., Armonk, NY) and all other analyses were using R statistical computing version 4.0.3 (R Foundation for Statistical Computing, Vienna, Austria.

Additional per-protocol analyses were performed with exclusion of patients that did not undergo allocated surgical treatment(van der Veen et al. J Clin Oncol. 2021;39(9):978–89). Furthermore, additional pain score analyses were performed, by using the mean of the first collected pain score of the day (generally collected during the morning rounds). In addition, pain scores were also analyzed by using the medians.


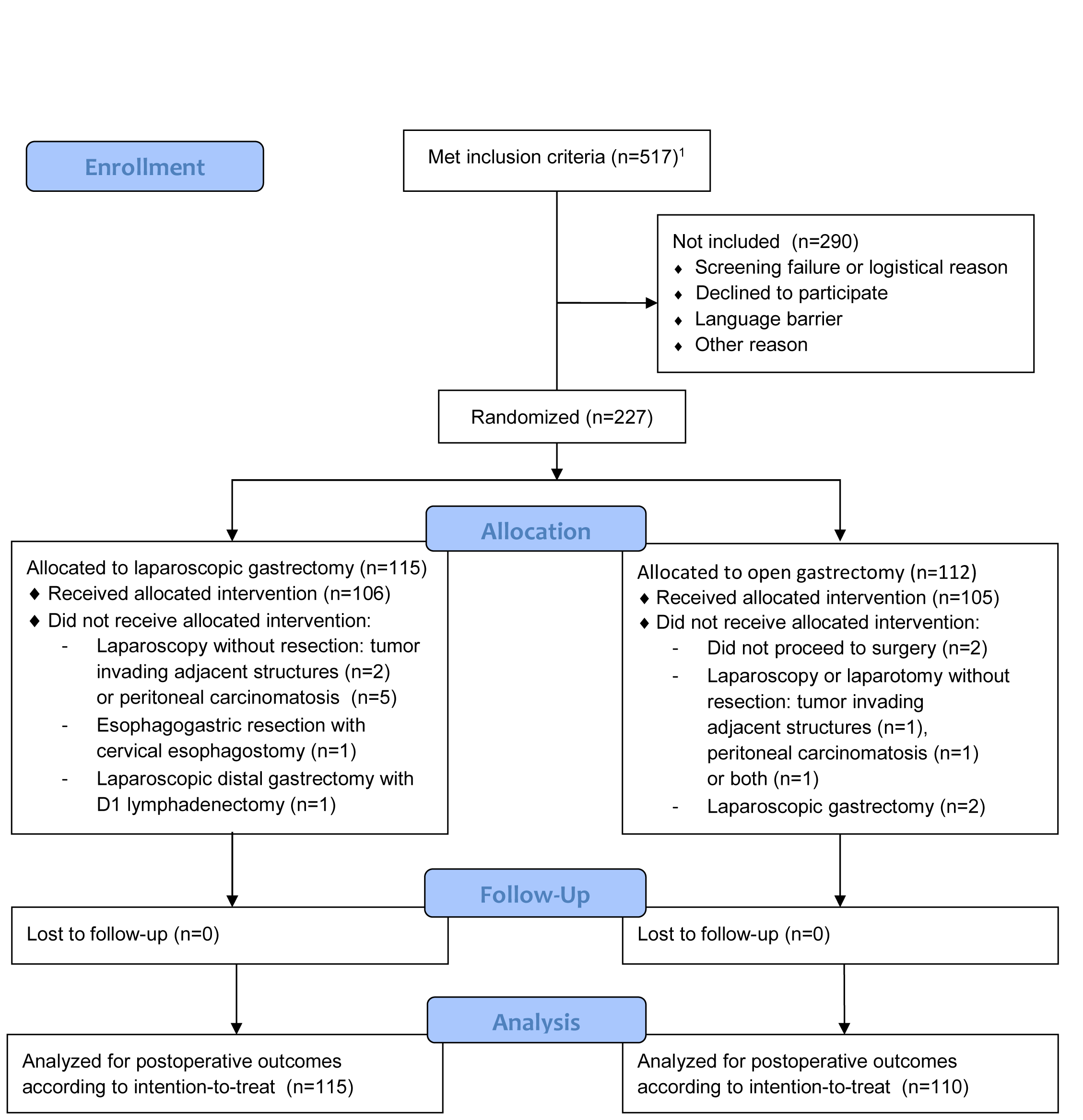


**Supplementary material 3** trial flow. A total of 225 patients who underwent randomization and surgery were included in the intention-to-treat analysis for postoperative outcomes: 115 in the laparoscopic gastrectomy group and 110 in the open gastrectomy group. A total of 211 patients underwent their allocated treatment according to protocol and were included in the per-protocol analyses: 106 in the laparoscopic gastrectomy group and 105 in the open gastrectomy group.

^1^The Dutch Upper GI Cancer Audit (DUCA) is a mandatory registration that contains every patient that underwent a gastrectomy for gastric cancer, including open-close procedures (Busweiler et al. Br J Surg. 2016;103(13):1855–63). DUCA data were used to calculate the total amount of patients that met the study inclusion criteria during the inclusion period of each trial center.

|  | Laparoscopic gastrectomy | | | | |  |  | Open gastrectomy | | | |  |
| --- | --- | --- | --- | --- | --- | --- | --- | --- | --- | --- | --- | --- |
|  | Epidural subgroup | | | |  |  |  | Epidural subgroup | | | |  |
| **n (%)** | n=16 | |  | *Missing or NA* | | |  | n=73 | |  | *Missing or NA* | |
| **Type of operation** |  |  |  | *0* | *(0)* |  |  |  |  |  | *0* | *(0)* |
| Total gastrectomy | 8 | ( 50.0) |  |  |  |  |  | 34 | ( 46.6) |  |  |  |
| Distal gastrectomy | 4 | ( 25.0) |  |  |  |  |  | 37 | ( 50.7) |  |  |  |
| Esophagogastric resection | 1 | ( 6.2) |  |  |  |  |  | 0 | ( 0.0) |  |  |  |
| No resection | 3 | ( 18.8) |  |  |  |  |  | 2 | ( 2.7) |  |  |  |
| **Epidural opioid type*** |  |  |  | *3* | *(18.8)* |  |  |  |  |  | *10* | *(13.7)* |
| Sufentanyl | 12 | ( 92.3) |  |  |  |  |  | 50 | ( 68.5) |  |  |  |
| Fentanyl | 0 | ( 0.0) |  |  |  |  |  | 3 | ( 4.1) |  |  |  |
| Morphine | 1 | ( 7.7) |  |  |  |  |  | 10 | ( 13.7) |  |  |  |
| **Epidural switched to local anesthetic only**** | 2 | ( 16.7) |  | 4 | *(25)* |  |  | 9 | ( 13.6) |  | *7* | *(9.6)* |
| **Epidural day of removal** |  |  |  | *0* | *(0)* |  |  |  |  |  | *0* | *(0)* |
| POD 0*** | 2 | ( 12.5) |  |  |  |  |  | 2 | ( 2.7) |  |  |  |
| POD 1 | 2 | ( 12.5) |  |  |  |  |  | 10 | ( 13.7) |  |  |  |
| POD 2 | 4 | ( 25.0) |  |  |  |  |  | 21 | ( 28.8) |  |  |  |
| POD 3 | 6 | ( 37.5) |  |  |  |  |  | 28 | ( 38.4) |  |  |  |
| POD 4 | 2 | ( 12.5) |  |  |  |  |  | 6 | ( 8.2) |  |  |  |
| POD 5 | 0 | ( 0.0) |  |  |  |  |  | 4 | ( 5.5) |  |  |  |
| POD 6 | 0 | ( 0.0) |  |  |  |  |  | 2 | ( 2.7) |  |  |  |
| **Epidural replacement** | 0 | ( 0.0) |  | *0* | *(0)* |  |  | 1 | ( 1.4) |  | *0* | *(0)* |
| **Epidural adequate sensibel block POD 1** | 13 | (100.0) |  | 3 | *(18.8)* |  |  | 65 | ( 97.0) |  | 6 | *(8.2)* |
| **Epidural adequate sensibel block POD 2** | 11 | ( 91.7) |  | *4* | *(25)* |  |  | 59 | ( 96.7) |  | *12* | *(16.4)* |
| **Epidural adequate sensibel block POD 3** | 7 | ( 77.8) |  | 7 | *(43.8)* |  |  | 39 | ( 95.1) |  | 32 | *(43.8)* |
| **Epidural adequate sensibel block POD 4** | 2 | (100.0) |  | 14 | *(87.5)* |  |  | 11 | (100.0) |  | 62 | *(84.9)* |
| **Epidural adequate sensibel block POD 5** | 0 | ( NA) |  | *16* | *(100)* |  |  | 5 | (100.0) |  | *68* | *(93.2)* |
| **Epidural top-up** |  |  |  | *4* | *(25)* |  |  |  |  |  | *11* | *(68.8)* |
| Performed once, effective | 1 | ( 8.3) |  |  |  |  |  | 3 | ( 4.8) |  |  |  |
| Performed once, not effective | 0 | ( 0.0) |  |  |  |  |  | 3 | ( 4.8) |  |  |  |
| Performed twice, both effective | 0 | ( 0.0) |  |  |  |  |  | 2 | ( 3.2) |  |  |  |
| Performed twice, once effective | 0 | ( 0.0) |  |  |  |  |  | 1 | ( 1.6) |  |  |  |
| Not performed | 11 | ( 91.7) |  |  |  |  |  | 53 | ( 85.5) |  |  |  |
| **IV opioid POD 1-5****** | 3 | ( 21.4) |  | 2 | *(12.5)* |  |  | 20 | ( 28.2) |  | *2* | *(2.7)* |
| **Epidural related complication** |  |  |  | 1 | *(6.3)* |  |  |  |  |  | *0* | *(0)* |
| Minor complication: hypotension | 0 | ( 0.0) |  |  |  |  |  | 6 | ( 8.2) |  |  |  |
| Major complication | 0 | ( 0.0) |  |  |  |  |  | 0 | ( 0.0) |  |  |  |
| None | 15 | (100.0) |  |  |  |  |  | 67 | ( 91.8) |  |  |  |
| **Supplementary material 4** epidural details. NA = not applicable; IV = intravenous; POD = postoperative day. *If epidural opioids were given, then the type of opioid was constant over de the different PODs.  ** This variable indicates whether at any time during POD 1-5 the opioid was removed from the epidural and thus only a local anesthetic was given over the epidural.  ***POD 0 = day of surgery. ****This variable indicates whether IV opioids were given at least once during the first 5 PODs. | | | | | | | | | | | | |


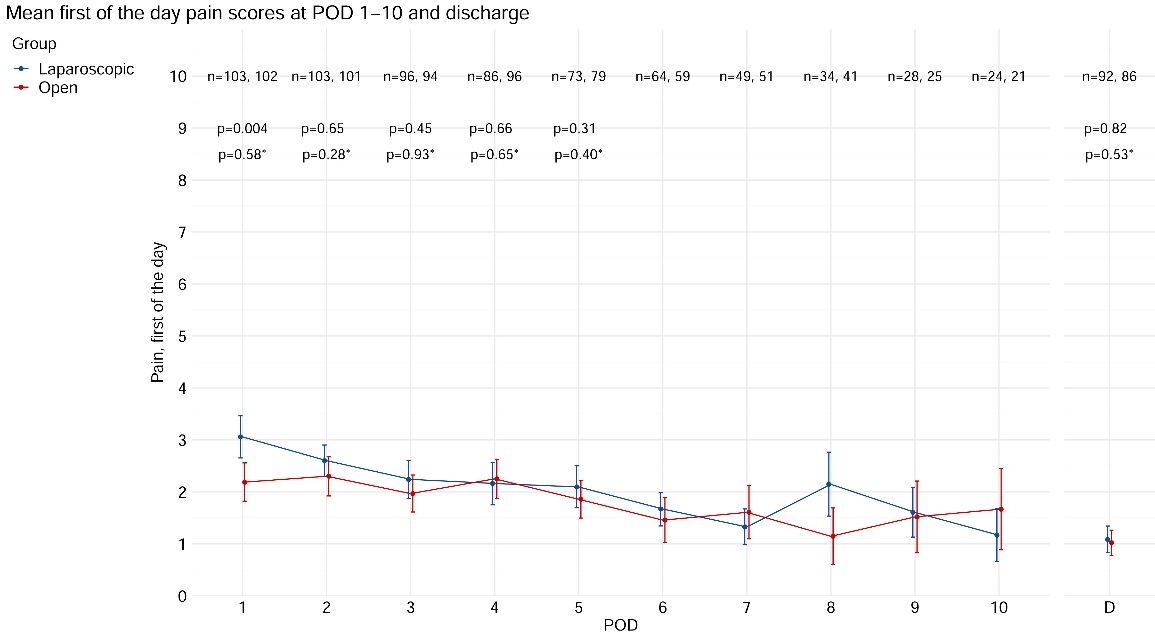


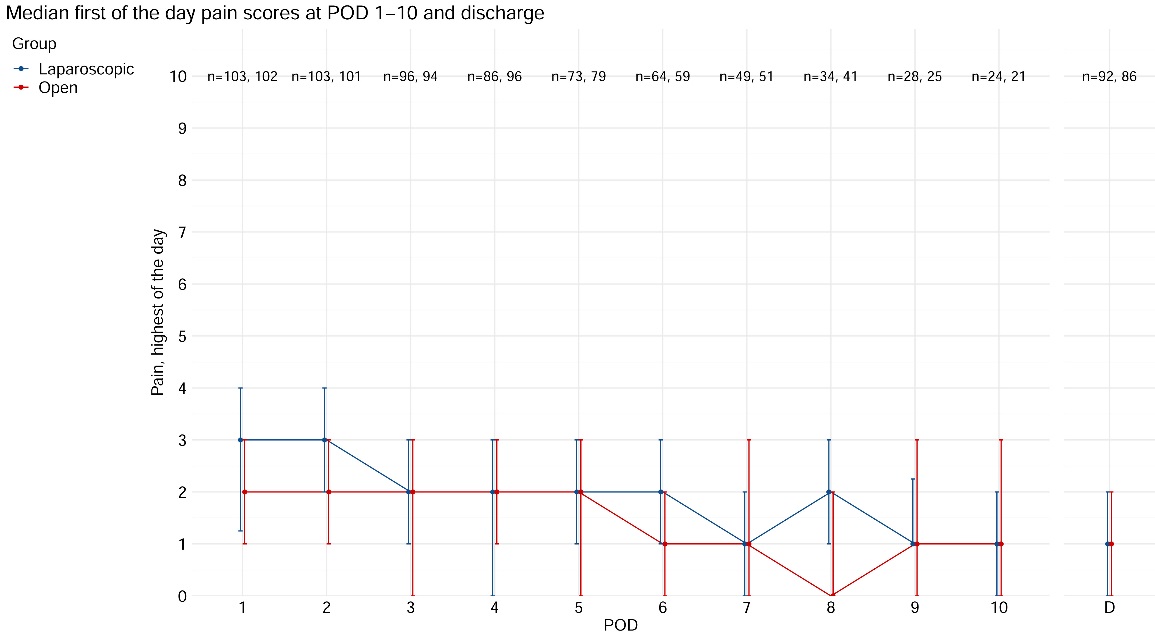


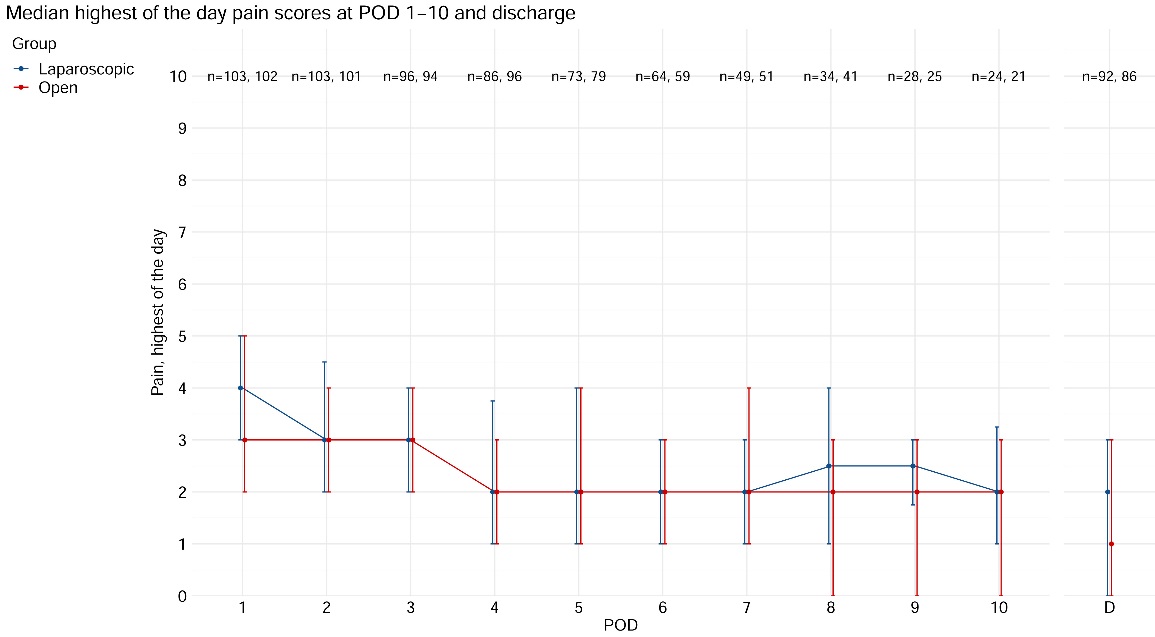


**Supplementary material 5** pain scores: mean first of the day, median first of the day and median highest of the day at POD 1-10 and discharge, with 95% confidence intervals for means and interquartile ranges for medians. P-values from de mixed model between group comparison at POD 1-5 and linear regression at discharge (table 3) are displayed above the brackets. * = p-value corrected for epidural analgesia. POD = Postoperative Day, D = Day of discharge. Of note, day of discharge is variable per patient and often not directly following POD 10.
