# Supplementary material for: Pain and Opioid Consumption After Laparoscopic Versus Open Gastrectomy for Gastric Cancer: A Secondary Analysis of a Multicenter Randomized Clinical Trial (LOGICA-Trial)
Source: J Gastrointest Surg. 2023 Jul 18;27(10):2057–67. doi: 10.1007/s11605-023-05728-3 (PMC10579125; doi:10.1007/s11605-023-05728-3)
Supplement: Supplementary file 2 — Supplementary file2 (PDF 1511 KB) [file 11605_2023_5728_MOESM2_ESM.pdf]

Laparoscopic versus Open Gastrectomy

–

A multicenter prospectively randomized controlled trial

March 2019

PROTOCOL TITLE

Laparoscopic versus **O**pen **G**astrectomy for Gastr**i**c **Ca**ncer - a multicenter prospectively randomized controlled trial

**Protocol ID**	-
**Short title**	LOGICA trial: Laparoscopic versus Open Gastrectomy for Gastric Cancer
**Version**	4.1
**Date**	06-03-2019
**Coordinating investigator(s)/project leader(s)**	Drs. A. van der Veen<br>a.vanderveen-12@umcutrecht.nl<br><br>Drs. H.J.F. Brenkman<br>h.j.f.brenkman@umcutrecht.nl<br>Drs. M.F.J. Seesing<br>m.f.j.seesing@umcutrecht.nl<br>Drs. L.Haverkamp<br>leonie.haverkamp@gmail.com<br><br>Dept. of Surgery, G04.228<br>University Medical Center Utrecht<br>Heidelberglaan 100, 3584 CX Utrecht, The Netherlands<br>T: +31 (0)88-755 8074 / F: +31 (0)30-254 1944
**Principal investigator(s) (in Dutch: hoofdonderzoeker/ uitvoerder)**	Prof. dr. R. van Hillegersberg<br>r.vanhillegersberg@umcutrecht.nl<br>Dr. J.P. Ruurda<br>j.p.ruurda@umcutrecht.nl<br><br>Dept. of Surgery, G04.228<br>University Medical Center Utrecht<br>Heidelberglaan 100, 3584 CX Utrecht, The Netherlands<br>T: +31 (0)88-755 8074 / F: +31 (0)30-254 1944
Multicenter research per site:	Academisch Medisch Centrum, Amsterdam<br>Dr. S.S. Gisbertz / S.S.Gisbertz@amc.uva.nl

	Catharina Ziekenhuis, Eindhoven<br>Dr. M. Luyer / misha.luyer@catharinaziekenhuis.nl<br><br>Erasmus Medisch Centrum, Rotterdam<br>Dr. B.P.L. Wijnhoven / B.Wijnhoven@erasmusmc.nl<br><br>Leids Universitair Medisch Centrum, Leiden<br>Dr. H.H. Hartgrink / H.H.Hartgrink@lumc.nl<br><br>Orbis Medisch Centrum, Sittard<br>Dr. J.H.M.B. Stoot / J.Stoot@orbisconcern.nl<br>Vrije Universiteit Medisch Centum, Amsterdam<br>Dr. D.L. van der Peet / dl.vanderpeet@vumc.nl<br><br>Zorggroep Twente, locatie Almelo<br>Dr. E.A. Kouwenhoven / E.Kouwenhoven@zgt.nl<br><br>Meander Medisch Centrum, Amersfoort<br>Prof. Dr. I.A.M.J. Broeders<br>IAMJ.Broeders@meandermc.nl<br><br>Gelre ziekenhuizen, Apeldoorn<br>Dr. E.B. Wassenaar, e.wassenaar@gelre.nl
Patient association:	N. Plum, Patiëntenperspectief Cancer Center UMCU<br>J.H.F. Leemhuis, SPKS, patiëntengroep Maagkanker
**Sponsor (in Dutch: verrichter/opdrachtgever)**	Prof. dr. R. van Hillegersberg<br>r.vanhillegersberg@umcutrecht.nl<br>Dept. of Surgery, G04.228<br>University Medical Center Utrecht<br>Heidelberglaan 100, 3584 CX Utrecht, The Netherlands<br>T: +31 (0)88-755 8074 / F: +31 (0)30-254 1944
**Independent expert**	Dr. L.M.G. Moons<br>Dept. of Hepatology and Gastroenterology, G04.228<br>University Medical Center Utrecht<br>Heidelberglaan 100, 3584 CX Utrecht, The Netherlands<br>T: +31 (0)88-755 5555 / L.M.G.Moons@umcutrecht.nl

TABLE OF CONTENTS

1. INTRODUCTION AND RATIONALE 7
2. OBJECTIVES 10
3. STUDY DESIGN 10
4. STUDY POPULATION 12
   4.1 Population (base) 13
   4.2 Inclusion criteria 13
   4.3 Exclusion criteria 14
   4.4 Sample size calculation 14
5. TREATMENT OF SUBJECTS 15
6. METHODS 18
   6.1 Study parameters/endpoints 18
      6.1.1 Main study parameter/endpoint 18
      6.1.2 Secondary study parameters/endpoints 19
      6.1.3 Other study parameters 19
   6.2 Randomisation, blinding and treatment allocation 20
   6.3 Study procedures 20
   6.4 Withdrawal of individual subjects 21
   6.5 Replacement of individual subjects after withdrawal 21
   6.6 Follow-up of subjects withdrawn from treatment 21
   6.7 Inclusion of additional subjects 21
7. SAFETY REPORTING 22
   7.1 Section 10 WMO event 22
   7.2 AEs, SAEs and SUSARs 22
      7.2.1 Adverse events (AEs) 22
      7.2.2 Serious adverse events (SAEs) 22
      7.2.3 Suspected unexpected serious adverse reactions (SUSARs) 23
   7.3 Annual safety report 23
   7.4 Follow-up of adverse events 23
   7.5 Data Safety Monitoring Board (DSMB) 23
8. STATISTICAL ANALYSIS 24
   8.1 Primary study parameter 24
   8.2 Secondary study parameters 24
   8.3 Other study parameters 25
   68.4 Interim analysis 25
9. ETHICAL CONSIDERATIONS 25
   9.1 Regulation statement 25
   9.2 Recruitment and consent 25
   9.3 Objection by minors or incapacitated subjects (if applicable) 26
   9.4 Benefits and risks assessment, group relatedness 26
   9.5 Compensation for injury 26
   9.6 Incentives (if applicable) 26

10. ADMINISTRATIVE ASPECTS, MONITORING AND PUBLICATION 27
10.1 Handling and storage of data and documents 27
10.2 Monitoring and Quality Assurance 27
10.3 Amendments 27
10.4 Annual progress report 27
10.5 End of study report 27
10.6 Public disclosure and publication policy 28
REFERENCES 29
11. Appendices 311
11.1 Appendix 1. Postoperative morbidity and mortality 311
11.2 Appendix 2. Quality of Life questionnaires 322
11.3 Appendix 3. VAS score 373
11.4 Appendix 4. SMEQ questionnaire 387
11.5 Appendix 5. Patient Information Brochure and Informed Consent form 38
11.6 Appendix 6. Groningen Frailty Indicator Score 40
11.7 Appendic 7.Short Nutritional Assessment Questionaire Score 42

SUMMARY

Rationale: Gastric cancer is a life-threatening disease with an incidence of 2.000 in the Netherlands. Surgical resection is the cornerstone of curative treatment. In the Netherlands this procedure is mainly performed by open surgery, whereas our recent meta-analysis of cohort studies showed that laparoscopic gastrectomy is associated with reduced intraoperative blood loss, reduced postoperative complications and shorter hospital stay. These benefits were at the cost of longer operative time. However, thus far all studies were performed in an Asian population containing a large percentage of T1-2 tumors. There is no RCT in a Western population with locally advanced gastric cancer. In the Netherlands experience with laparoscopic gastrectomy has increased in the last years, with now more than 5 centers performing this type of operation on a routine basis. We have trained this technique to 30 surgeons visiting our Minimally Invasive Gastrectomy course in the past 2 years.

Objective: To evaluate the benefits, risks and costs of laparoscopic gastrectomy as an alternative to open gastrectomy (conventional surgery) as treatment for gastric cancer in a Western population.

Study Design: Multicenter prospectively randomized controlled trial.

Study population: Patients (age ≥ 18) with histological proven surgically resectable (cT1-4a, N0-3b, M0) gastric cancer with European Clinical Oncology Group (ECOG) performance status 0, 1 or 2.

Intervention: In total 210 patients will be randomized over 2 treatment groups; The experimental group consists of 105 patients that receive laparoscopic gastrectomy and the control consists of 105 patients that undergo conventional open gastrectomy.

Main study parameters/endpoints: Primary outcome is postoperative hospital stay (days). Secondary outcomes are cost-effectiveness, oncologic outcomes, postoperative morbidity, mortality, readmissions and quality of life.

Follow-up: 60 months after surgery

Nature and extent of the burden and risks associated with participation, benefit and group relatedness: Patients will undergo either laparoscopic gastrectomy (experimental arm) or the conventional open gastrectomy (conventional surgery). Tumor biopsies will be collected pre-operatively. Blood samples will be drawn at baseline, direct postoperatively and on postoperative day 2. Patients are required to fill out quality of life questionnaires (preoperatively and <5 days, 6 weeks, 6 months, and yearly up to 5 years postoperatively). Laparoscopic gastrectomy was demonstrated to be safe in our own series of 30 patients treated in the UMC Utrecht as well as in our recent meta-analysis of the literature.

1. INTRODUCTION AND RATIONALE

Gastric cancer treatment

Gastric cancer is the fourth most prevalent malignancy worldwide (1). In the Netherlands approximately 2.000 patients are newly diagnosed every year (2). Half of these patients are eligible for surgery. The 5-year survival rate is 45% after curative resection (3). Perioperative chemotherapy is now the standard of care as it has shown to increases 5-year survival with another 10% (4).

Surgery

In 1993, a laparoscopic approach to the treatment of gastric cancer was reported for the first time (5). Ever since, laparoscopic techniques and surgical experience have improved, allowing complex laparoscopic procedures such as laparoscopic distal gastrectomy and laparoscopic total gastrectomy to be performed. Systematic reviews and meta-analyses have shown an advantage in short-term outcomes of laparoscopic distal gastrectomy compared with open distal gastrectomy (6-11). Our recent meta-analysis, containing 8 comparative cohort studies, shows similar advantages for laparoscopic total gastrectomy (12).

Laparoscopic total gastrectomy was associated with a reduction of intra-operative blood loss (weighted mean difference 228 ml; 95% c.i. 311-144; $p<0.001$), a reduced risk at postoperative complications (risk ratio 0.51; 95% c.i. 0.33-0.77), and admission time was shortened from 18 top 14 days (figure 1, weighted mean difference 4 days; 95%c.i. 1.3-6.5; $p<0.001$). These benefits were at the cost of a prolonged duration of surgery (weighted mean difference 56 minutes (95% c.i. 25-86); $p< 0,001$). In hospital mortality rates were comparable for the laparoscopic (1%) and open approach (2%) (risk ratio 0.68; 95% c.i. 0.20-2.36).

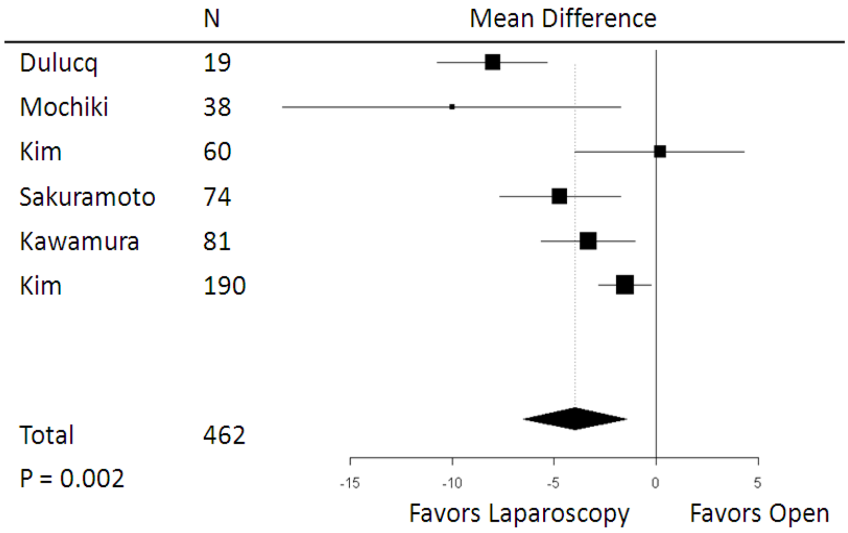

Figure 1: Postoperative hospital stay was evaluated in 7 out of 8 comparative cohort studies in our meta-analysis.[16] Hospital stay was shortened from 18 days after open gastrectomy to 14 days after laparoscopic gastrectomy.

This meta-analysis was predominantly based on patients from Asian studies (90%), since research on this topic is still limited in Western countries. Therefore, we analysed our own data in a Dutch cohort at the University Medical Center Utrecht of 30 patients treated between 2005-2013 for gastric cancer with laparoscopic versus open total gastrectomy (table 1. We found that blood loss was 265 (30-2700) ml for laparoscopic resection, compared to 613 (100-3000) ml for open resection (p=0.008). The duration of surgery was 4:57 (2:40-7:40) hours in the laparoscopic group, compared to 4:00 (2:40-6:20) hours in the open group (p=0.003). The median hospital stay was 12 days after laparoscopic total gastrectomy, versus 25 days after open total gastrectomy (p<0.001). We have trained this technique to 30 Dutch surgeons visiting our Minimally Invasive Gastrectomy course in the past 2 years. In the Netherlands experience with laparoscopic gastrectomy has therefore increased in the last 2 years, with now more than 5 centers performing this type of operation on a routine basis. Our 3rd course will be organized in March 2014.

No randomized clinical trial on laparoscopic gastrectomy has been performed in a Western country such as the Netherlands. The aim of the current study is to prospectively compare laparoscopic versus open gastrectomy in patients with gastric cancer by means of a randomized controlled trial.

	**Open Total gastrectomy N=24**	**Laparoscopic Total gastrectomy N=30**	**P-value**
Duration of surgery (h)*	4:00 (2:40-6:20)	4:57 (2:40-7:40)	0.003
Blood loss (ml)*	613 (100-3000)	265 (30-2700)	0.008
Conversion	-	8 (27%)	-
Complicated course	14 (58%)	16 (53%)	0.713
Postoperative bleeding	1 (4%)	2 (7%)	0.690
Anastomotic leakage	11 (46%)	5 (17%)	0.020
Anastomotic stricture	7 (29%)	6 (20%)	0.434
Dilated	7 (29%)	6 (20%)	0.434
Stent	6 (25%)	2 (7%)	0.590
Chylothorax	1 (4%)	1 (3%)	0.872
Postoperative hospital stay (d)*	25 (7-188)	12 (7-53)	0.003
Intensive care stay (d)*	5 (0-57)	1 (0-26)	0.020
Resected lymphnodes (n)*	16 (3-33)	19 (2-53)	0.376
Tumor positive (n)*	3 (0-15)	1 (0-33)	0.927
R0 resection	22 (92%)	28 (93%)	0.816
Mortality			
30-day mortality	1 (4.2%)	1 (3.3%)	0.087
60-day mortality	4 (16.7%)	2 (6.7%)	0.245

Table 1: Intraoperative and Postoperative Outcomes
***Results presented as medians (min-max)**
Reasons for conversion: tumor ingrowth in surrounding structures (6x), arterial bleeding (1x), venous bleeding (1). R0 resection: proximal and distal resection margin.

2. OBJECTIVES

The aim of this study is to prospectively compare laparoscopic versus open gastrectomy in patients with gastric cancer by means of a randomized controlled trial.

Primary outcome measure:

- Postoperative hospital stay

Secondary outcome measures:

- Cost-effectiveness
- Oncologic outcome
- Postoperative morbidity and mortality
- Quality of Life
- Readmissions

3. STUDY DESIGN

Design

The study design is a non-blinded multicenter prospectively randomized controlled trial. Analysis will take place on an intention-to-treat basis.

The participating patients are randomized for one of the 2 treatment arms. The experimental arm consists of the laparoscopic approach, whereas the control arm is the comparison to the conventional surgery, consisting of the open approach (figure 2).

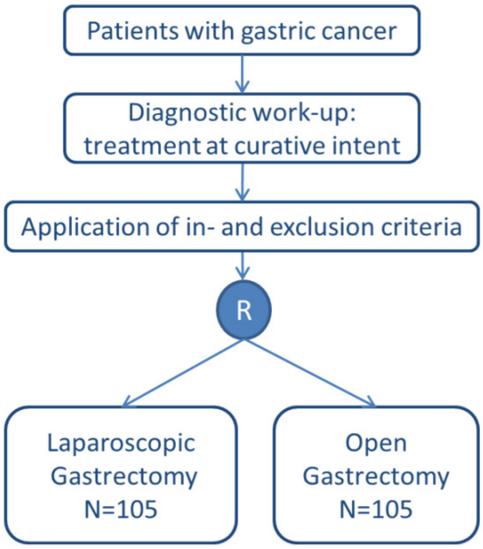

Figure 2: Treatment allocation. Based on tumor location patients will undergo either distal or total gastrectomy, according to the Dutch gastric cancer guidelines.

Duration

The duration of the study will be 3 years of inclusion + 5 years follow-up.

Setting

This study takes place in a hospital setting. Recruitment of patients will occur at the outpatient clinic. Participating patients will receive either laparoscopic (experimental arm) or open (conventional surgery) gastrectomy at the surgical theater. Postoperative care takes place at the surgical ward of the participating hospitals.

Quality of Life

The regular postoperative care for patients that undergo a gastrectomy for gastric cancer consists of an annual outpatient visit for the follow-up period of 5 years. This visit is used to check for signs of recurrence of disease and metastases, as well as long term complications. In addition to this standard care quality of life questionnaires will be sent to the patients home address to check the long term effects of the surgical procedure.

Cost-effectiveness

To evaluate the cost-effectiveness of both study procedures, an important secondary study parameter, medical costs will be related to productivity and quality of life. All medical costs related to the study procedure will be collected up to five years after the operation. Quality of life will be measured as mentioned above. Furthermore, study participants will be asked to fill in the questionnaire SF-HLQ to measure the productivity after surgery (appendix 3). An expert from the UMC Utrecht Julius Center will do the final cost-effectiveness analysis.

Blood samples

Blood samples will be drawn at baseline, 6 hours postoperatively and on postoperative day 2. The aim is to monitor the CRP, leukocytes, haemoglobin and a panel of interleukines production to obtain an indication of the inflammatory response during the early postoperative course.

Biobanking

As part of a biobank side study the patients will be asked to donate body material. This will include blood samples (2x 10 ml), and tumor tissue (4x 0,5 cm^3). The blood samples will be obtained during regular preoperative diagnostics and daily on the day of surgery until the fifth day after surgery. These extra blood samples will be analyzed on a broad panel of markers which may predict anastomic leakage in an early state. The tumor tissue will be obtained by

the pathologist after tumor resection. After informed consent, these materials will be stored for an indefinite time in the UMC Utrecht Biobank for future studies on biomarkers and genetics to predict disease progress.

Overview of activities

	Outpatient clinic	Surgery (day 0)	Hospitalization	Follow-up (6 weeks, 6, 12, 24, 36, 48, 60 months)
Demographic data	X			
Medical history	X			
Diagnostic work-up	X	-	-	-
Quality of Life assessment	X	-	X	X
VAS-score	X	-	X	X
Surgical details	-	X	-	-
Morbidity	-	X	X	X
Hospital stay	-	X	X	X
Pathological evaluation	-	X	X	-
Readmissions	-	-	-	X
Mortality	-	X	X	X

In addition to the follow-up moments mentioned in the table above, one quality of life questionnaire, the productivity questionnaire and the health care use questionnaire (appendix 3) will be sent 3 months and 9 months after surgery for a complete cost-effectiveness analysis.

4. STUDY POPULATION

4.1 Population (base)

Patients (≥18 years) who are diagnosed with gastric adenocarcinoma are eligible to participate in the study. The centres perform 20-30 gastric resections per year. A total number of 210 patients is required for inclusion, which makes the inclusion time of 3 years feasible.

4.2 Inclusion criteria

In order to be eligible to participate in this study, a subject must meet all of the following criteria:

- Histologically proven gastric adenocarcinoma
- Surgically resectable (cT1-4a, N0-3b, M0) tumor
- Age ≥ 18
- European Clinical Oncology Group (ECOG) performance status 0,1 or 2
- Written informed consent

T – Primairy tumor	
**T1a**	Lamina propria
**T1b**	Submucosa
**T2**	Muscularis propria
**T3**	Subserosa
**T4a**	Perforates serosa
**T4b**	Adjacent structures

N – Regional lymph nodes	
**N0**	No regional lymph node metastasis
**N1**	1-2 lymph node metastasis
**N2**	3-6 lymph node metastasis
**N3a**	7-15 lymph node metastasis
**N3b**	≥16 lymph node metastasis

M – Distant metastasis	
**M0**	No distant metastasis
**M1**	Distant metastasis

TNM7 classification UICC, www.uicc.org

4.3 Exclusion criteria

A potential subject who meets any of the following criteria will be excluded from participation in this study:

- Siewert type I junction tumor (tumor located between 1 and 5 cm proximal from the esophagogastric junction)
- Pregnant women
- Non-elective surgery
- Previous gastric resection or recurrent gastric cancer

4.4 Sample size calculation

The primary outcome parameter is postoperative hospital stay. A recent meta-analysis has shown that laparoscopic procedure shortens the median hospital stay with 4 days (12).
Hospital stay after laparoscopic gastrectomy: 14 days
Hospital stay after open gastrectomy: 18 days

Significance level (α) = 0.05
Power (1-β) = 0.80

Sample size:
Total number of 210 participants:

- Laparoscopic gastrectomy (experimental arm) = 105
- Open gastrectomy (conventional surgery) = 105

5. TREATMENT OF SUBJECTS

The investigational surgery is the laparoscopic gastrectomy (distal and total). The conventional surgery treatment consists of conventional open gastrectomy (distal and total). Both techniques are described underneath.

Patients will receive a distal gastrectomy or total gastrectomy, depending on tumor location on preoperative diagnostics (Endoscopic ultrasonography [EUS], gastroscopy and CT-scan). For tumors located in the antrum a distal gastrectomy will be performed, whereas for tumors located in the corpus, and diffuse gastric cancer, a total gastrectomy will be performed (13).

Basic summary of the surgical procedure:

- Distal gastrectomy with D2 lymph node dissection (figure 3)
- Total gastrectomy with D2 lymph node dissection (figure 3)

Several types of reconstruction types can be performed (i.e. jejunal pouch or no pouch), according to the preference of the surgeon. Also the type of anastomosis (i.e. end-to-end, end-to-side or side-to-side) either stapled or hand sewn may be performed according to the individual surgeon's insight.

Distal gastrectomy	Total gastrectomy
D1: Station 1,3,4sb, 4d, 5, 6, 7<br>D1+: Station 1,3,4sb, 4d, 5, 6, 7, 8a, 9<br>D2: Station 1,3,4sb, 4d, 5, 6, 7, 8a, 9, 11p, 12a	D1: Station 1-7<br>D1+: Station 1-7, 8a, 9, 11p<br>D2: Station 1-7, 8a, 9, 11p, 11d, 12a
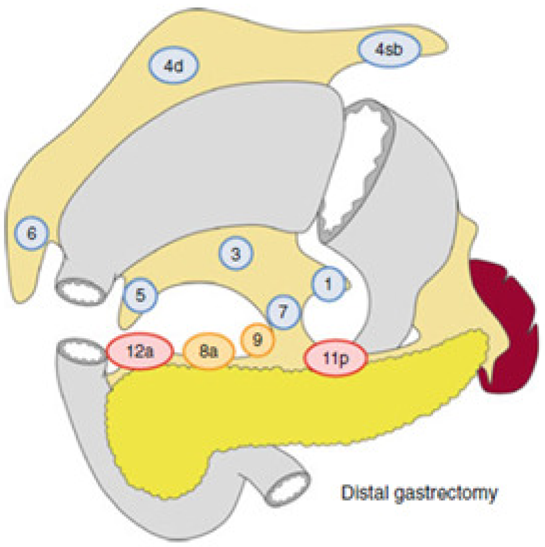	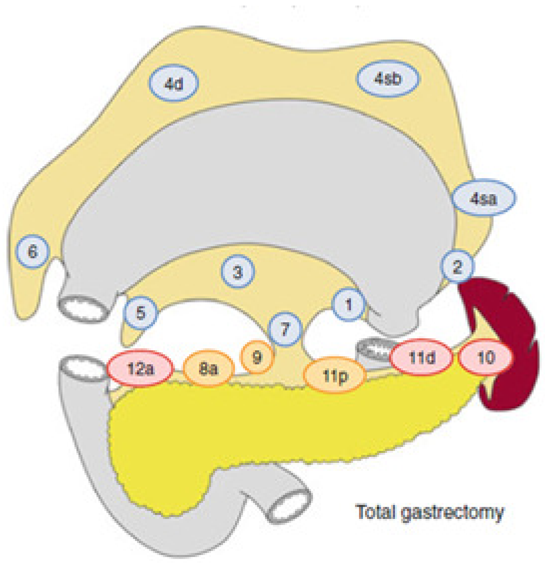

Figure 3: lymph node dissection for gastrectomy, based on Japanese gastric cancer treatment guidelines 2010 (ver. 3)

<u>Incision:</u>

- The incisions have to be muscle-sparing. The length of the mini-laparotomy (≤5 cm), for the laparoscopic procedure, and the length of the laporotomy for the open procedure, have to be noted on the CRF.
- Maximally 5 trocar ports are used (figure 4).

Surgical Quality Control

A selected number of trained surgeons will perform both types of surgery. Hereby we maintain quality of the operations and minimize differences in success rates for both groups. The participating centers have completed their learning curves (n≥20) for laparoscopic gastrectomy, since this may affect morbidity and mortality.

It is of great importance that all participating surgeons perform both the open and the laparoscopic procedure in a similar fashion. To ensure this, all selected surgeons participated in the course "*One day course on minimally invasive gastrectomy*", which is organized at the UMC Utrecht. The yearly course has been given at 01-11-2012 and 27-06-2013, and is also scheduled for March 2014. During the course an extensive explanation of the laparoscopic technique is combined with practical hands-on gastrectomy session on human cadavers. The surgeons are instructed by the principal investigators (RvH and JR) of this study.

To ensure consistency in techniques between the participating centers, a proctor will supervise, or systematically review the videos of 2 2 laparoscopic proceduresfrom all centers.

Total gastrectomy:

The patient is positioned in supine position under general anaesthesia. The conventional open total gastrectomy is performed by means of an upper midline laparotomy. In case of the laparoscopic procedure, 5 ports are used: a camera port (10mm) , two working ports (5mm) , an assisting port (5mm) and a port (12mm) for the liver retractor. Placement of all ports is done according to the surgeons preference. The minor and greater curvatures of the stomach are dissected. The left gastric artery and vein are transected at their origin. Next, the right gastroepiploic artery and the right gastric artery are transected at their origin. The postpyloric duodenum is cleaved by means of a linear endostapler. Subsequently, the distal esophagus is dissected from the left and right crus and mobilized. At the distal esophagus 2 support sutures are placed to pull the esophagus into the abdominal cavity, after which the distal oesophagus is cleaved. Frozen section histology of the tumor resection planes is performed to assess the radicality of the resection. Optional frozen section histology can be performed for detection of local tumor invasion and metastases. The greater omentum is resected for future side-studies. Subsequently, a transverse incision is performed to take out the stomach and greater omentum (figure 1, A). A hand-sewn jejuno-jejunostomy is created. Next, the actual esophagojejunostomy is performed. The formation of a jejunal pouch and the placement of a feeding jejunostomy should be performed according to the local hospital's policy.

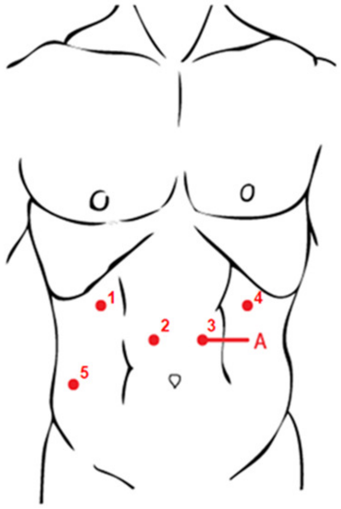

Figure 4: Maximally 5 trocar ports are used. Trocar positions: trocar 1 (5mm), 2 (5mm), and 4 (5mm) are used for instrumentation used during the LTG. The camera is inserted through trocar 3 (10mm), which is the location of the minilaparotomy (A) for the removal of the stomach as well. The liver retractor is inserted through trocar 5 (12mm).

Distal gastrectomy

The conventional open distal gastrectomy is performed by means of a midline laparotomy. For laparoscopy, first a pneumoperitoneum is obtained by introducing the first trocar under direct visual control. This trocar is placed in the umbilicus. The next four trocars can be placed according to the surgeon's preference. The minor omentum is opened. Next, the greater curvature of the stomach is prepared. The gastrocolic ligament is divided at 3 cm distal to the gastroepiploic artery, after which the greater curvature is skeletonized up to the gastrosplenic ligament. The right gastroepiploic vein and artery are clipped at its origin. Next the right gastric vessels are clipped. The duodenum is divided distally at 1 cm distal to the pyloric sphincter. The proximal side of the stomach is divided at least 6cm above the tumor. Partial omentectomy is performed in the area of the antrum. The removal of the resected specimen occurs via a mini-laparotomy (max. 5-6 cm), which can be located according to the surgeon's insight. The minilaparotomy must be muscle sparing as describes above. Finally, a Roux-en-Y gastrojejunostomy is performed.

Lymphadenectomy

Lymph node dissection will be performed based on the *Dutch oncologic guidelines* and *Japanese gastric cancer treatment guidelines* (13-15). For D2 lymphadenectomy no pancreato-splenectomy is performed since this is associated with high post-operative morbidity and mortality (3). Furthermore, lymph node station 10 is not dissected during total gastrectomy since it is technically difficult and associated with poor prognosis when affected (3,13). Lymph node stations 1, 3, 4d, 4sb, 5-9, 11p and 12a are dissected during distal gastrectomy. Lymph node stations 1-3, 4d, 4sa, 4sb, 5-9, 11p, 11d and 12a are dissected

during total gastrectomy. All lymph node stations will be marked by the surgical team and presented to the pathologist for evaluation.

Diagnostics:

- Gastroesophagoscopyand biopsy for histopathologic analysis (PA).
- Endoscopic ultrasonography.
- CT-abdomen.
- CT-thorax.

Preoperative policy

- Preoperative nutrition will be given to patients with >10% weight loss the last 6 months or a BMI <18kg/cm^2, according to the 2009 European Society of Clinical Nutriorion and Metabolism (ESPEN) guidelines (16) .

Postoperative policy

To ensure fast recovery, the ERAS protocol is followed. A consensus meeting between the participating centers is organized to discuss the pre- and postoperative protocol(17).

- Epidural analgesia will be given following open gastrectomy only. Other analgesia can be given without limitations to reach adequate pain control.
- Urinary catheterization can be done when indicated.
- Enteral feeding via a jejunostomy catheter can be given to patients who underwent total gastrectomy, according to the local hospital's policy. No jejunostomy catheter will be placed following distal gastrectomy.
- At postoperative day 1, patients will be encouraged to start liquid oral feeding.
- Mobilization under supervision of a physiotherapist starts as soon the patient is stable.
- Nasojejunal tubes for decompression will not be used (17).

However, the treating physician may implement a deviation from the protocol to avoid causing any harm or if there is a safety hazard to trial subjects without prior approval from the ethics committee.

6. METHODS

6.1 Study parameters/endpoints

6.1.1 Main study parameter/endpoint

- Postoperative hospital stay (days)

  The following criteria have to be met:

  - Adequate pain control with oral pain medication

- Started with mobilisation
- Adequate intake according to nutritional demand and being independent of supplementary intravenous fluids

6.1.2 Secondary study parameters/endpoints

- Oncologic outcome:
  - Resection margins in millimeters
  - R0 resection (complete resection with no microscopic residual tumor)
  - Number of lymph nodes dissected
  - Number of tumor positive lymph nodes dissected
- Postoperative morbidity and mortality (19) (see appendix 1)
- Quality of Life: EQ-5D-5L, EORTC QLQ-30, EORTC QLQ-STO22 (see appendix 2)
- Cost-effectiveness: (see appendix 3)
  - SF-HLQ
  - Health care use
  - Costs of the procedure, hospital stay and re-interventions
  - Post-operative quality of life and productivity
- Readmission

6.1.3 Other study parameters

Baseline characteristics:

- Gender
- Age
- ASA-classification
- Body Mass Index
- Comorbidities
- Sarcopenia (see appendix 8)
- Intoxications
- Perioperative chemotherapy

Postoperative outcomes:

- Operation characteristics:
  - Duration of surgery
  - Blood loss during operation
  - Conversion to alternative surgical procedure

- Postoperative course:
  - Duration of Intensive Care stay
  - Duration of Medium Care stay
  - Mechanical ventilation
  - Vital signs (heart rate, blood pressure, temperature)
- Overall survival
- Disease free survival (absence of local or distant recurrence)
- Patients experience:
  - Pain: VAS-score (see appendix 4)
  - Time to return to daily activity (days)
  - Time to return to normal nutritional regime (days)
- Weight at day 5 after surgery or at discharge in case of discharge before day 5.
- Ergonomics surgeon: SMEQ questionnaire (see appendix 5)
- Learning curves of participating centers

6.2 Randomisation, blinding and treatment allocation

After completion of preoperative chemotherapy and signing informed consent at the outpatient clinic, participants will be randomized by means of an online random number generator, stratified by centre. Randomization will be performed by the coordinating investigator and directly provided to the participating centres. The data-analyst will be blinded for the procedure.

6.3 Study procedures

Patient will undergo one of the following surgical procedures:

Laparoscopic gastrectomy: n= 105
Open gastrectomy: n= 105

Additional measures:

**Pre-operative**	**Post-operative**
Blood sample for biobanking and baseline	Blood 6h postoperatively and on postoperative day 2.
	VAS-score for pain and vital signs during hospital stay
QoL assessment<br>Groningen Frailty Indicator Score<br>SNAQ score	QoL assessment and VAS scores for pain at 6 weeks, 3, 6, 9 monhts and 1-5 year after surgery.<br>Productivity assessment (SF-HLQ questionnaire)<br>Health care use (questionnaire appendix 3)

6.4 Withdrawal of individual subjects

Subjects can leave the study at any time for any reason if they wish to do so without any consequences. The investigator can decide to withdraw a subject from the study for urgent medical reasons. Subjects not undergoing the allocated treatment (e.g. intraoperatively unresectable tumor) due to medical reasons will not be withdrawn from the study unless the subject requests to do so. Participants will be analysed in an intention-to-treat model.

6.5 Replacement of individual subjects after withdrawal

Study participants will be replaced by newly recruited and randomized subjects in case of withdrawal before surgery, in order to meet the sample size requirement. Patients who withdraw after hospital discharge will not be replaced.

6.6 Follow-up of subjects withdrawn from treatment

Patients who withdraw from the study will be followed-up until moment of withdrawal.

6.7 Inclusion of additional patients

For subjects not undergoing the allocated treatment (e.g. unresectable tumor) due to medical reasons, additional subjects will be included in the study to reach the calculated sample size (n=210).

7. SAFETY REPORTING

7.1 Section 10 WMO event

In accordance to section 10, subsection 1, of the WMO, the investigator will inform the subjects and the reviewing accredited METC if anything occurs, on the basis of which it appears that the disadvantages of participation may be significantly greater than was foreseen in the research proposal. The study will be suspended pending further review by the accredited METC, except insofar as suspension would jeopardise the subjects' health. The investigator will take care that all subjects are kept informed.

7.2 AEs, SAEs and SUSARs

7.2.1 Adverse events (AEs)

Adverse events are defined as any undesirable experience occurring to a subject during the study, whether or not considered related to allocated surgery. All adverse events of a modified Clavien-Dindo classification grade 2 and higher and reported spontaneously by the subject or observed by the investigator or his staff, will be recorded starting from the day of surgery until the start of adjuvant therapy or up to 3 months after surgery. Events occurring outside of this period are not expected to be related to the study intervention, but more likely to (neo)adjuvant treatment or disease progression.

7.2.2 Serious adverse events (SAEs)

A serious adverse event is any untoward medical occurrence or effect that at any dose:

- results in death;
- is life threatening (at the time of the event);
- requires hospitalisation or prolongation of existing inpatients' hospitalisation;
- results in persistent or significant disability or incapacity;
- is a congenital anomaly or birth defect;
- Any other important medical event that may not result in death, be life threatening, or require hospitalization, may be considered a serious adverse experience when, based upon appropriate medical judgement, the event may jeopardize the subject or may require an intervention to prevent one of the outcomes listed above.

The SAE's that will be reported during this study are SAE's occurring starting from the day of surgery until the start of adjuvant therapy or up to 3 months after surgery.

Events occurring outside of this period are not expected to be related to the study intervention, but more likely to (neo)adjuvant treatment or disease progression.
The local coordinating investigator per centre is responsible for reporting the SAEs to the sponsor.

The sponsor will report the SAEs through the web portal *ToetsingOnline* to the accredited METC that approved the protocol, within 3 months after the sponsor has first knowledge of the serious adverse reactions by means of a periodical overview

SAEs that result in death should be reported expedited. The expedited reporting will occur not later than 7 days after the responsible investigator has first knowledge of the adverse reaction. This is for a preliminary report with another 8 days for completion of the report.

7.2.3 Suspected unexpected serious adverse reactions (SUSARs)

Not applicable.

7.3 Annual safety report

Not applicable.

7.4 Follow-up of adverse events

All AEs will be followed until they have abated, or until a stable situation has been reached. Depending on the event, follow up may require additional tests or medical procedures as indicated, and/or referral to the general physician or a medical specialist.
SAEs need to be reported till end of study within the Netherlands, as defined in the protocol

7.5 Data Safety Monitoring Board (DSMB)

The stop-criteria are:

- <70% complete resection without tumor residual in the proximal or distal surgical resection plane (R0) in one or both arms,
- >50% less than 10 lymph node harvested in one or both arms.

A data safety monitoring board will be assigned to evaluate the outcomes at 105 patients.

A DSMB is established to perform ongoing safety surveillance and to perform interim analyses on the safety data. This committee is an independent committee, which is composed of (names will be determined at a later stage).
None of the members have a conflict of interest with the sponsor of the study.

Criteria on which the DSMB may decide to terminate the trial prematurely:

- Clear evidence of benefit of laparoscopic gastrectomy
- Clear evidence of harm of laparoscopic gastrectomy
- External evidence, such as other trials or published data, not available during the start of this trial.

The advice(s) of the DSMB will only be sent to the sponsor of the study. Should the sponsor decide not to fully implement the advice of the DSMB, the sponsor will send the advice to the reviewing METC, including a note to substantiate why (part of) the advice of the DSMB will not be followed.

8. STATISTICAL ANALYSIS

Subjects will be analysed by means of an intention-to-treat model.

8.1 Primary study parameter

The primary outcome measure is postoperative hospital stay. This variable is expected to have a non-Gaussian distribution and will therefore be analysed by means of a Mann-Whitney-U test in univariate analysis.

8.2 Secondary study parameters

Binary variables will be displayed in cross-tabulations. The Person χ^2 will be used for analysis between groups.

Variables with a non-Gaussian distribution and will be analysed by means of a Mann-Whitney-U test in univariate analysis.

Statistical significance is defined as $P<0.05$.

8.3 Other study parameters

Binary variables will be displayed in cross-tabulations. The Person χ^2 will be used for analysis between groups.

Variables with a non-Gaussian distribution and will be analysed by means of a Mann-Whitney-U test in univariate analysis.

Statistical significance is defined as P<0.05.

8.4 Interim analysis

If the DSMB requires an interim-analysis for benefit the Peto approach will be followed meaning that the study will only be stopped for beneficial effects in case of a P<0.001. An interim analysis is performed at 105 patients. The interim-analysis is performed by the study coordinator and the epidemiologist/statistician of the DSMB together and discussed in a private meeting by only the DSMB members. If the DSMB suspects harm there will be a meeting between the DSMB, the trial research group and an independent statistician. During this meeting there should be a discussion about any potential causal relation between gastrectomy and harm based on the current literature. The final decision is made by the DSMB and their opinion is sent to the study coordinator and principal investigator. There will be no futility for this trial. The ethics committee will receive a direct copy of this advice.

9. ETHICAL CONSIDERATIONS

9.1 Regulation statement

This study will be conducted according to the principles of the Declaration of Helsinki (2008) and in accordance with the Medical Research Involving Human Subjects Act (WMO).

9.2 Recruitment and consent

Patients are recruited to participate in this trial at the outpatient clinic. They are introduced to the study by a sub-investigator who will not be involved in the care and treatment of the patient but who is part of the study team and therefore can inform the patient about the study and answer the questions of the patient. The subjects will be fully informed about the study by means of a patient information letter that explains the trial. Potential participants can contact the coordinating investigator or independent physician for any questions concerning the trial. The subjects will be given a week to consider their

decision. The informed consent form will be signed by both the participant and the investigator. The patient information brochure and the informed consent form are attached (appendix 5)

9.3 Objection by minors or incapacitated subjects (if applicable)

Not applicable

9.4 Benefits and risks assessment, group relatedness

All surgical techniques used in this research are known to be safe.

9.5 Compensation for injury

The sponsor/investigator obtained dispensation from the statutory obligation to provide insurance from the Medical Ethics Committee UMC Utrecht. The reason for dispensation is that if a study includes two different, but commonly used procedures, the insurance does not cover damage caused by the study.

9.6 Incentives (if applicable)

Not applicable

10. ADMINISTRATIVE ASPECTS, MONITORING AND PUBLICATION

10.1 Handling and storage of data and documents

The subjects will be coded by patient number in order to handle personal information about patients anonymously. The patient's information will solely be available to this research group by the use of SPSS guarded with a password. The coordinating investigator and the principal investigator know this password. The data will be kept for 15 years. This will be done in concordance with the Dutch Personal Data Protection Act (Wet Bescherming Persoonsgegevens, WBP)

10.2 Monitoring and Quality Assurance

The study is classified as a low risk study (verwaarloosbaar risico) and will be monitored accordingly. For details, see monitoring plan.

10.3 Amendments

Amendments are changes made to the research after a favourable opinion by the accredited METC has been given. All amendments will be notified to the METC that gave a favourable opinion.

10.4 Annual progress report

The investigator will submit a summary of the progress of the trial to the accredited METC once a year. Information will be provided on the date of inclusion of the first subject, numbers of subjects included and numbers of subjects that have completed the trial, serious adverse events/ serious adverse reactions, other problems, and amendments.

10.5 End of study report

The investigator will notify the accredited METC of the end of the study within a period of 8 weeks. The end of the study is defined as the last patient's last visit.

In case the study is ended prematurely, the investigator will notify the accredited METC within 15 days, including the reasons for the premature termination.

Within one year after the end of the study, the investigator/sponsor will submit a final study report with the results of the study, including any publications/abstracts of the study, to the accredited METC.

10.6 Public disclosure and publication policy

Not applicable

REFERENCES

(1) Parkin DM, Bray F, Ferlay J, Pisani P. Global cancer statistics, 2002. CA Cancer J Clin 2005 Mar-Apr;55(2):74-108.

(2) Poos M, van Kranen H, Wijnhoven B. Hoe vaak komt maagkanker voor en hoeveel mensen sterven eraan? 2010.

(3) Hartgrink HH, van de Velde CJ, Putter H, Bonenkamp JJ, Klein Kranenbarg E, Songun I, et al. Extended lymph node dissection for gastric cancer: who may benefit? Final results of the randomized Dutch gastric cancer group trial. J Clin Oncol 2004 Jun 1;22(11):2069-2077.

(4) Cunningham D, Allum WH, Stenning SP, Thompson JN, Van de Velde CJ, Nicolson M, et al. Perioperative chemotherapy versus surgery alone for resectable gastroesophageal cancer. N Engl J Med 2006 Jul 6;355(1):11-20.

(5) Kitano S, Iso Y, Moriyama M, Sugimachi K. Laparoscopy-assisted Billroth I gastrectomy. Surg Laparosc Endosc 1994 Apr;4(2):146-148.

(6) Memon MA, Subramanya MS, Khan S, Hossain MB, Osland E, Memon B. Meta-analysis of D1 versus D2 gastrectomy for gastric adenocarcinoma. Ann Surg 2011 May;253(5):900-911.

(7) Ohtani H, Tamamori Y, Noguchi K, Azuma T, Fujimoto S, Oba H, et al. Meta-analysis of laparoscopy-assisted and open distal gastrectomy for gastric cancer. J Surg Res 2011 Dec;171(2):479-485.

(8) Martinez-Ramos D, Miralles-Tena JM, Cuesta MA, Escrig-Sos J, Van der Peet D, Hoashi JS, et al. Laparoscopy versus open surgery for advanced and resectable gastric cancer: a meta-analysis. Rev Esp Enferm Dig 2011 Mar;103(3):133-141.

(9) Chen XZ, Hu JK. Meta-analysis of laparoscopic and open distal gastrectomy for gastric carcinoma. Surg Endosc 2009 May;23(5):1156-1157.

(10) Kodera Y, Fujiwara M, Ohashi N, Nakayama G, Koike M, Morita S, et al. Laparoscopic surgery for gastric cancer: a collective review with meta-analysis of randomized trials. J Am Coll Surg 2010 Nov;211(5):677-686.

(11) Hosono S, Arimoto Y, Ohtani H, Kanamiya Y. Meta-analysis of short-term outcomes after laparoscopy-assisted distal gastrectomy. World J Gastroenterol 2006 Dec 21;12(47):7676-7683.

(12) Haverkamp L, Weijs TJ, van der Sluis PC, van der Tweel I, Ruurda JP, van Hillegersberg R. Laparoscopic total gastrectomy versus open total gastrectomy for cancer: a systematic review and meta-analysis. Surg Endosc 2013 May;27(5):1509-1520.

(13) Oncoline. Landelijke werkgroep Gastro-intestinale Tumoren Versie: 1.0. 2009; Available at: http://oncoline.nl/maagcarcinoom.

(14) Japanese Gastric Cancer Association. Japanese gastric cancer treatment guidelines 2010 (ver. 3). Gastric Cancer 2011 Jun;14(2):113-123.

(15) Lee JH, Lee HJ, Kong SH, Park do J, Lee HS, Kim WH, et al. Analysis of the lymphatic stream to predict sentinel nodes in gastric cancer patients. Ann Surg Oncol 2014 Apr;21(4):1090-1098.

(16) Braga M, Ljungqvist O, Soeters P, Fearon K, Weimann A, Bozzetti F, et al. ESPEN Guidelines on Parenteral Nutrition: surgery. Clin Nutr 2009 Aug;28(4):378-386.

(17) Mortensen K, Nilsson M, Slim K, Schafer M, Mariette C, Braga M, et al. Consensus guidelines for enhanced recovery after gastrectomy: Enhanced Recovery After Surgery (ERAS(R)) Society recommendations. Br J Surg 2014 Jul 21.

(18) Wang Z, Chen J, Su K, Dong Z. Abdominal drainage versus no drainage post gastrectomy for gastric cancer. Cochrane Database Syst Rev 2011 Aug 10;(8):CD008788. doi(8):CD008788.

(19) Low DE, Alderson D, Cecconello I, Chang AC, Darling GE, D'Journo XB, et al. International Consensus on Standardization of Data Collection for Complications Associated With Esophagectomy: Esophagectomy Complications Consensus Group (ECCG). Ann Surg 2015 Jan 20.

11. Appendices

11.1 Appendix 1 Postoperative morbidity and mortality

- Anastomotic leakage
- Anastomotic stricture
- Respiratory complications
- Cardiac complications
- Intra-abdominal bleeding
- Intra-abdominal abscess
- Sepsis
- Ileus
- Wound infection
- Fistula
- Urinary tract infection
- Dumping syndrome
- Mortality

All complications will be defined according to the Esophageal Complications Consensus Group. All complications will be scored according to the modified Clavien-Dindo classification and the Comprehensive Complication Index.

11.2 Appendix 2 Quality of Life questionnaires

- EQ-5D-5L
- EORTC QLQ-30
- EORTC QLQ-STO22

11.3 Appendix 3 Cost-effectiveness questionairres

- SF-HLQ

<u>Instructie</u>

Deze vragenlijst gaat over de gevolgen van gezondheidsproblemen voor betaald werk en ontbetaald werk (bijv. huishoudelijk werk). Deze vragen hebben steeds betrekking op de afgelopen 4 weken. Met gezondheidsproblemen worden zowel uw lichamelijke als uw emotionele problemen bedoeld.

1. Hebt u momenteel betaald werk?

   ☐ Nee *(gaat u door naar vraag 9)*

   ☐ Ja

Voor hoeveel uur per week hebt u een aanstelling? ………… uren per week

Over hoeveel dagen zijn deze uren verdeeld? ………… dagen

Wat is uw beroep? ……………………………………

2. Hebt u de afgelopen 4 weken verzuimd van betaald werk vanwege gezondheidsproblemen?

   ☐ Nee

   ☐ Ja, ik heb ………… werkdagen verzuimd

   ☐ Ja, ik heb ………… werkdagen verzuimd, maar ik heb hiervan wel …. dagen op arbeidstherapeutische basis gewerkt

Hebt u langer dan de gehele afgelopen 4 weken verzuimd van betaald werk vanwege gezondheidsproblemen?

☐ Nee

☐ Ja, ik heb me ziek gemeld sinds ……………………………*(gaat u door naar vraag 8)*

Mensen met gezondheidsproblemen moeten daarvoor soms verzuimen van hun werk. Het kan echter voorkomen dat iemand **wel** op zijn werk aanwezig is (eventueel op arbeidstherapeutische basis), maar zijn werk minder goed doet vanwege gezondheidsproblemen, daarover gaan de vragen 3 t/m 7.

3. Werd u de afgelopen 4 weken op uw betaald werk gehinderd door gezondheidsproblemen?

   ☐ Nee, in het geheel niet *(gaat u door naar vraag 6)*

   ☐ Ja, een beetje

   ☐ Ja, heel erg

4. Hoeveel dagen in de afgelopen 4 weken hebt u wel betaald werk verricht (eventueel op arbeidstherapeutische basis), terwijl u last had van gezondheidsproblemen?

   ………… dagen (*de dagen waarop u helemaal niet hebt gewerkt, omdat u zich ziek hebt gemeld hoeft u **niet** mee te rekenen)*

5. Wilt u aangeven hoe goed u hebt gewerkt op de dagen waarop u wel op uw werk was terwijl u last had van gezondheidsproblemen.
   (Een 1 betekent dat u zeer slecht in staat was uw werk uit te voeren en een 10 betekent dat uw werk niet werd beïnvloed.)

1	2	3	4	5	6	7	8	9	10
☐	☐	☐	☐	☐	☐	☐	☐	☐	☐
zeer slecht									even goed als normaal

6. Nu volgt een aantal uitspraken die voor mensen met gezondheidsproblemen van toepassing kunnen zijn met betrekking tot betaald werk. Geef aan hoe vaak elke uitspraak op u van toepassing was in de afgelopen 4 weken

   Ik was wel op mijn werk, maar als gevolg van gezondheidsproblemen ……

	bijna nooit	soms	vaak	bijna altijd
had ik concentratiestoornissen	☐	☐	☐	☐
moest ik in een langzamer tempo werken	☐	☐	☐	☐
moest ik mij afzonderen	☐	☐	☐	☐
had ik meer problemen om beslissingen te nemen	☐	☐	☐	☐
moest ik werk uitstellen	☐	☐	☐	☐
moest ik werk laten overnemen door anderen	☐	☐	☐	☐
had ik andere problemen, nl: ……………………………………	☐	☐	☐	☐

7. Als u het werk zou moeten inhalen dat u in de afgelopen 4 weken niet hebt kunnen verrichten vanwege uw gezondheidsproblemen, hoeveel uur zou u dan moeten werken?

   ………… uur (*de dagen waarop u helemaal niet hebt gewerkt, omdat u zich ziek hebt gemeld hoeft u **niet** mee te rekenen)*

8. Wat is uw eigen netto inkomen uit betaald werk?
   (Het gaat om het gedrag dat u 'schoon' in uw handen krijgt. Voor alle duidelijkheid: het gaat alleen om uw eigen inkomen, dus zonder dat van uw eventuele partner)

   ☐ € ………… per week

   ☐ € ………… per 4 weken

   ☐ € ………… per maand

   ☐ € ………… per jaar

   ☐ Weet ik niet / wil ik niet zeggen

9. Welke van de volgende situaties is op u van toepassing? Indien meerdere situaties van toepassing zijn, wilt u dan aangeven welke situatie het meest op u van toepassing is.

   ☐ Ik heb een betaalde baan

   ☐ Ik zorg voor het huishouden (en eventueel kinderen)

   ☐ Ik ben gepensioneerd of met prepensioen

   ☐ Ik ben scholier of student

   ☐ Ik kan (gedeeltelijk) geen betaald werk doen vanwege gezondheidsproblemen en ben voor …………..% arbeidsongeschikt

   ☐ Ik doe geen betaald werk om andere redenen
   (Bijv. vanwege onvrijwillige werkloosheid of vrijwilligerswerk)

10. Hebben anderen in de afgelopen 4 weken huishoudelijke taken die u normaal wel doet overgenomen in verband met gezondheidsproblemen?

    ☐ Nee

    ☐ Ja, namelijk (meerdere antwoorden mogelijk)
    - o gezinsleden voor ……… uur
    - o andere onbetaalde mensen voor ……… uur
    - o thuiszorg voor ……… uur
    - o andere betaalde hulp voor ……… uur

- Health care use questionnaire

NL47444.041.14 LOGICA trial www.logica-trial.nl

Vragenlijst zorggebuik

Hoe vaak bent u de **afgelopen 6 weken**:

1. bij de huisarts geweest? ……. maal
2. op de spoedeisende hulp of huisartsenpost geweest? ……. maal
3. op de polikliniek in het ziekenhuis geweest? ……. maal
4. opgenomen geweest in het ziekenhuis? ……. maal
   a. Hoeveel dagen was dit in het totaal? ……. dagen

Waar verbleef u de **afgelopen 6 weken**:

5. Thuis: Niet / Altijd / …. Dagen
6. Revalidatieplek Niet / Altijd / …. Dagen
7. Verpleeghuis Niet / Altijd / …. Dagen
8. Overig, namelijk:
   a. ……………………………… …. Dagen
   b. ……………………………… …. Dagen
   c. ……………………………… …. Dagen

Heeft u de **afgelopen 6 weken:**

9. Hulp in huis gehad van thuis-, buurt- of mantelzorg Nee / Ja,
   a. Indien ja, hoevaak? …. maal per week / dag

Appendix 4 VAS score

Belasting van de operatie:

1. Kunt op deze schaal aangeven hoeveel pijn u nu heeft?
U doet dat door een kruisje (X) te zetten tussen geen pijn en heel veel pijn.

Geen pijn ____ Heel veel pijn

2. Kunt u op deze schaal aangeven hoeveel pijn u nu heeft ter plaatse van de operatiewonden?
U doet dat door een kruisje (X) te zetten tussen geen pijn en heel veel pijn.

Geen pijn ____ Heel veel pijn

3. Hoe hinderlijk is nu voor u de pijn van de operatiewonden?
Graag het hokje van uw keuze aankruisen

□ niet
□ een beetje
□ nogal
□ heel erg

4. Wij willen graag weten hoe belastend, al met al, de behandeling voor u is geweest? Zou u op de lijn hieronder willen aangeven hoe belastend u het vond?
U doet dat door een kruisje (X) te zetten tussen helemaal niet belastend en heel erg belastend.

Helemaal niet belastend ____ Heel erg belastend

5. In hoeverre vindt u dat de behandeling de moeite waar is geweest?
Graag het hokje van uw keuze aankruisen

□ niet
□ een beetje
□ nogal
□ heel erg

6. Indien u nu voor de keuze zou staan, zou u dan voor dezelfde behandeling kiezen?
Graag het hokje van uw keuze aankruisen.

□ ja
□ nee
□ ik weet niet

Zo nee, kunt u aangeven waarom niet? ……………………………………………………………

………

11.4 Appendix 5 SMEQ questionnaire

This graphic displays the amount of effort it took you to execute the task you have been working on.

Please score the amount of effort by marking one of the anchors on the vertical line here below.

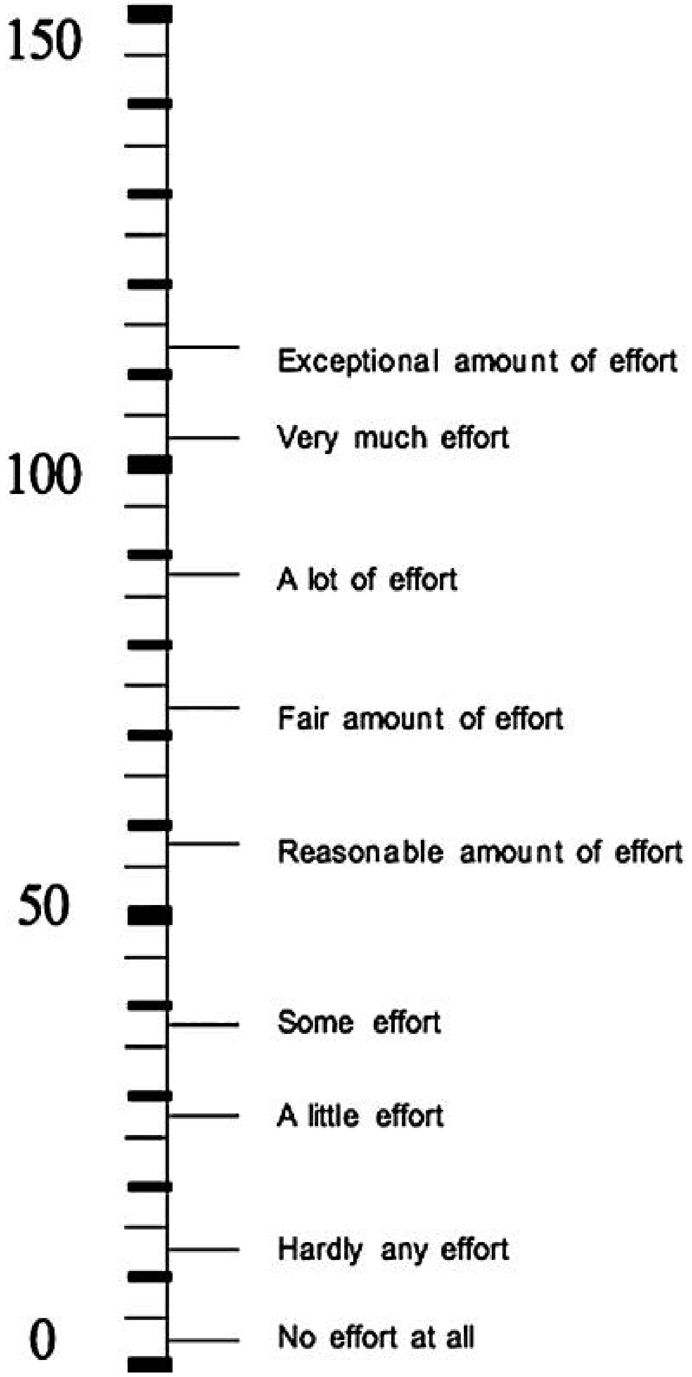

11.5 Appendix 5 Patient Information Brochure and Informed Consent form

- Patient Information Brochure LOGICA-trial
- Informed Consent form LOGICA-trial
- Patient Information Brochure Biobanking
- Informed Consent form Biobanking

11.6 Appendix 6

Items of Groningen Frailty Indicator Score

Mobility

Is the patient able to carry out these tasks single-handed without any help? (The use of help resources, such as walking stick, walking frame, wheelchair, is considered independent.)

1. Shopping
2. Walking around outside (around the house or to the neighbors)
3. Dressing and undressing
4. Going to the toilet

Physical Fitness

5. What mark does the patient give himself/herself for physical fitness? (scale 0-10)

Vision

6. Does the patient experience problems in daily life due to poor vision?

Hearing

7. Does the patient experience problems in daily life due to being hard of hearing?

Nourishment

8. During the last 6 months has the patient lost a lot of weight unwillingly? (3 kg in 1 month or 6 kg in 2 months)

Morbidity

9. Does the patient take 4 or more different types of medicine?

Cognition (Perception)

10. Does the patient have any complaints about his/her memory or is the patient known to have a dementia syndrome?

Psychosocial

11. Does the patient sometimes experience emptiness around him/ her?

12. Does the patient sometimes miss people around him/her?

13. Does the patient sometimes feel abandoned?

14. Has the patient recently felt downhearted or sad?

15. Has the patient recently felt nervous or anxious?

Sum

Scoring:

Questions 1–4: Independent = 0; dependent = 1

Question 5: 0–6 = 1; 7–10 = 0

Questions 6–9: No = 0; yes = 1

Question 10: No and sometimes = 0; yes = 1

Questions 11–15: No = 0; sometimes and yes = 1

11.7 Appendix 7

Items of Short Nutritional Assessment Questionnaire Score

Item	Score
1.Did you lose weight unintentionally?	
-More than 6 kg in the last 6 months	3
- More than 3 kg in the last month	2
Did you experience a decreased appetite over the last month?	1
Did you use supplemental drinks or tube feeding over the last month?	1

11.8 Appendix 8

Sarcopenia will be assessed on the preoperative CT scans (these scans were made as standard of care) by the study team of the Orbis Medisch Centrum in Sittard. The cross sectional skeletal muscle surface (cm^2) assessment of sarcopenia will be performed at the level of the third lumbar vertebra (L3) on a single slice transversal coupe on which both vertebral spines are visible. This measurements will be performed using Slice-O-Matic software. Each participating centre will use the study subject ID to pseudonymise the CT-scans of their study patients and transfer the scans to the study team of the Orbis Medisch Centrum. The Orbis Medisch Centrum cannot trace the study subject ID back to individual patient identities and the patient privacy is thus protected. The transfer of the scans will be performed according to secured localized procedures of each centre.
